# A comparative study on culture-specific and cross-cultural aspects of intercultural relations in Hungary, Serbia, Czech Republic, and Germany

**DOI:** 10.3389/fpsyg.2022.886100

**Published:** 2022-10-06

**Authors:** Petia Genkova, Jonathan Herbst, Henrik Schreiber, Martina Rašticová, Jozsef Poor, Klara Valentinyi Veresné, Csilla Suhajda, Andrea Viszetenvelt, Jovana Bjekic

**Affiliations:** ^1^Department of Social and Economic Sciences, Osnabrück University of Applied Sciences, Osnabrück, Germany; ^2^Department of Law and Social Sciences, Mendel University in Brno, Brno, Czechia; ^3^Social Sciences Management and HR, Research Center, Hungarian University of Agriculture and Life Sciences, Gödöllö, Hungary; ^4^J. Selye University, Kománo, Slovakia; ^5^Institute for Medical Research, University of Belgrade, Belgrade, Serbia

**Keywords:** ethnic identity, prejudice, acculturation, cultural intelligence, multicultural personality, measurement equivalence

## Abstract

The ability, will, and belief that it is possible to deal effectively with members of other cultural/ethnic groups are still gaining importance all over the world. However, the majority of studies on intercultural relations focus on Western Europe and the USA, applying constructs and theories that replicate a western-centered worldview. As a consequence, it is unclear whether established measures for intergroup attitudes and intercultural competence may be applied in Eastern European countries and to what extent they display comparable ideas, thoughts, and feelings. The current study thus explores cross-cultural commonalities and differences in established measures of ethnic identity, prejudice, acculturation strategies, intercultural intelligence, and multicultural personality. Therefore, we compare the scale structure, difficulty, and sensitivity in samples from Germany and the Eastern European countries Hungary, Serbia, and the Czech Republic (etic-perspective), as well as the culture-specific conceptions of said concepts (emic-perspective). Results show that the investigated scales do not work comparably across German and Eastern European samples. Differences might be rooted in variations of underlying thinking patterns and connotations of single expressions. Those variations are likely to be related to the constant individual societal and historical developments of cultures, shaping the way individuals think and talk about cultural diversity. Future studies are encouraged to consider culture-specific and generalizable aspects of constructs when conducting cross-cultural research on intercultural relations.

## Introduction

One of the aims of social psychology and related fields is to understand and improve relations between cultural, ethnical, and national groups. During the past 30 years, researchers investigated thoroughly how certain constructs predict positive/negative intergroup attitudes and behavior (e.g., Pettigrew, 1998; Guimond et al., 2014). Promising ways to increase intergroup relations in culturally heterogeneous groups refer to cultural majority members ethnic identity, prejudice, acculturation-strategies, and dimensions of competence in intercultural interaction, i.e., intercultural intelligence and multicultural personality (Sims and Killen, 2020). While research on and measures for these constructs mostly stem from western cultures, researchers apply them to Eastern European settings more and more (Barzykowski et al., 2019; Olejárová et al., 2020). However, studies on culture-specific and cross-cultural aspects of intergroup relations referring to factor structure and linguistic idiosyncrasies are scarce (Bene, 2018). As a consequence, it is unclear to what extent established measures for intergroup attitudes and intercultural competence display comparable ideas, thoughts, and feelings. In order to further examine intergroup relations in Eastern Europe, careful analysis of culture-specific and cross-cultural aspects of intergroup relations is required. Therefore, we analyzed factor structures and item characteristics of scales for five important intergroup relation constructs (ethnic identity, subtle and blatant prejudices, acculturation strategies, intercultural intelligence, and multicultural personality) among samples from cultural majority groups in Germany, Czech Republic, Hungary, and Serbia. Previous analysis of the relationship between intercultural competence and prejudice revealed violations of measurement invariance but did not address the identification and interpretation of cross-cultural differences in relation to culture-specific results appropriately (Genkova et al., 2021). In the current study, we thus conducted secondary-data-analysis to explore in greater detail which constructs, dimensions, or single aspects concur or differ across the samples and discuss potential relations to cultural idiosyncrasies. Thereby, we aim to contribute to the understanding of intergroup relations in an international research context.

## Theoretical background

This study is based on a universalist paradigm on cross-cultural research, assuming that culture is a system for psychological orientation and belonging (Thomas and Simon, 2007) that influences the development, expression, and connotation of underlying, generalizable constructs and processes (see also Genkova, 2012; 2019; 2021). Previous research on intergroup relationships in different countries supports this perspective, indicating that underlying cognitive functions, e.g., categorization of social environment into social groups, are likely to work equivalently across cultures. However, connotations and forms of expression are rooted in the cultural context, e.g., the form and meaning of ethnicity. Accordingly, Guimond et al. (2014) argue that it “seems reasonable to consider that prejudice may result from the operation of processes that are found in certain cultural settings but not in others (i.e., emic), in addition to processes that are found to be common across cultures (i.e., etic)” (p. 942). Assuming that the perception of oneself and others as well as the perception of categorization and identification are fundamental for intergroup relationships in each culture (Yuki, 2003; Hamamura, 2017), one of the core predictors of intergroup relations should be ethnic identity.

### Ethnic identity

Ethnic identity is one major aspect of one’s social identity and is seen as a cognitive precondition for the development of interethnic/-cultural attitudes. According to the social identity approach, one’s identity is comprised of the categorization of the self and others toward social groups based on subjectively important characteristics, such as ethnicity (Tajfel and Turner, 1979; Park and Judd, 2005). Even though ethnicity is connotated and interpreted differently in each ethnic or cultural group, a recent study on the cross-cultural equivalence of identification of self and others showed that the individually perceived importance of ethnicity moderated the relationship of ethnic identification and interethnic bias comparably across 12.810 participants from 103 social groups (Grigoryan et al., 2022). Studies using the well-established assessment tool for ethnic identity, the Multigroup Ethnic Identity Measure (MEIM) by Phinney (1992), however, show inconsistent results. Building on previous research by Tajfel and Turner (1979) and Marcia (1980), the MEIM assesses ethnic identity by means of three core components that were assumed to be common across ethnic groups: a sense of belonging, a developmental concept, and involvement in ethnic practices (Phinney and Ong, 2007). Phinney (1992) also included a measure of attitudes toward ethnic outgroups. Previous studies found a clear distinctiveness of belonging to the ethnic ingroup and to ethnic outgroups as well as results supporting a one-factor solution for belonging to the ingroup (e.g., Ponterotto et al., 2003; Schachner et al., 2016). Phinney (1992) suggested that three components explain one overall factor of identity. In contrast, Ashmore et al. (2004) emphasize the need to consider the multidimensionality of ethnic identity. However, it is still not clear, which aspects of identity occur in Eastern European countries, which aspects have “value and emotional significance” (Tajfel, 1981, p. 255), and how they might be interrelated (Yuki, 2003). A two-factor solution (exploration and commitment) was found more fitting across samples from several Eastern European cultures (Ukrainians, ethnic minorities in USA, Czechs, Bulgarians; Roberts et al., 1999; Ganeva and Rasticova, 2013; Navarro et al., 2020). However, those studies did not consider differences in understanding due to, e.g., language idiosyncrasies (Fisher et al., 2020). To contribute to the understanding of meaning and connotation of what is seen as aspects of ethnic identity by Phinney (1992), the current study explores the factor structure of the MEIM plus the belonging to the outgroup scale (Phinney, 1992), exploring differences in factor structure, difficulty, and sensitivity.

### Prejudices and acculturation strategies

Prejudices are defined as negative primarily affective attitudes toward a social group, which often lead to negative intergroup behavior (Pettigrew and Meertens, 1995). It is at least questionable, to what extent cognitive representations of in- and outgroup identification are comparable across cultures (Yuki, 2003). The same might apply to prejudices linked to these representations, their connotation, and related social norms. To account for the declining social acceptance of hostile attitudes, researchers refer to the blatant and subtle prejudice scale (BSP) by Pettigrew and Meertens (1995). Blatant prejudices describe an openly expressed hostility and include threat/rejection and opposition to intimacy. Belief in traditional values, emphasizing cultural differences, and seemingly positive emotions form subtle prejudices, which describe a covert and socially more acceptable but nevertheless devaluating form of hostility. While Zick et al. (2008) describe the successful application of this scale in western European countries, validations of specific translated versions for Eastern European countries are scarce. Instead, studies from Eastern European countries focused on anti-Roma-attitudes (Kende et al., 2021), or blatant and subtle dehumanization of immigrants, especially refugees, since 2015 (Kteily et al., 2015; Bruneau et al., 2018). The content and connotation of prejudices against foreigners are thus very likely to depend on the cultural context (history of immigration policies for example).

Closely related to attitudes toward the in- and outgroup are acculturation strategies. Acculturation strategies are defined as behaviors and attitudes related to the mutual acculturation of interaction partners in prolonged intercultural contact situations, such as in multicultural societies (Berry, 1992, 2016). The concept of acculturation strategies was introduced by Berry (1992), who differentiated between individuals, which want to discard or maintain migrants’ heritage culture, and individuals who prefer contact between cultural groups. Dependent on the constellation of those attitudes, the four possible acculturation strategies integration, assimilation, separation, and marginalization, emerge. While acculturation strategies initially described reactions toward immigration strongly related to the own identity, Berry later referred to acculturation strategies (i.e., when talking about host-culture members) as more normative attitudes about how host- and guest-culture members should interact (Berry, 2001, 2016; Verkuyten, 2005, 2011; Verkuyten and Martinovic, 2006). In this study, the 13-item scale by van Dick et al. (1997) is applied. The acculturation strategy marginalization is not included in this scale, as it was considered less relevant in preceding studies (Berry, 1992). The scale was developed among Canadian samples but has been applied all over the world ever since. A cross-cultural comparison found evidence of validity for the underlying concept of acculturation in samples from Germany, Turkey, Switzerland, Slovakia, and former Yugoslavia, but not for a Hungarian sample (Piontkowski et al., 2000). Explorative factor analysis of the acculturation scale revealed cross-cultural differences in the affiliation of single items, suggesting that understanding of the items varies between cultures and languages, as well as between members of the culturally dominant group and non-dominant group (Piontkowski et al., 2000). Therefore, the meaning and connotation of items for acculturation strategies appear to be culture-specific.

### Intercultural competence

In addition to the question of attitudes toward immigration, there is considerable evidence that individuals differ not only in their willingness but also their ability to interact with members of other cultures. This ability to interact appropriately and efficiently with people from different cultures is called intercultural competence (Thomas and Simon, 2007; Deardorff, 2009). While there are numerous models of intercultural competence (for an overview, see, e.g., Genkova, 2019), two concepts are currently receiving more attention than others and are rather well grounded in theory, namely intercultural intelligence and multicultural personality.

Intercultural intelligence was conceptualized by Earley and Ang (2003) as a multilayered ability that interferes with social and emotional intelligence (based on the idea of multiple intelligences by Gardner, 1985). Van Dyne et al. (2008) further developed the model by adding a meta-cognitive component and providing the cultural intelligence scale (CQS), one of the most widespread measurements for intercultural competence. The scale assesses four independent facets of intercultural intelligence: Cognitive (knowledge), motivational (strife to interact), behavioral (adaption of behavior), and meta-cognitive (reflection and anticipation) competence. Thereby, Van Dyne et al. (2008) strived to provide a generalizable scale in order to predict intercultural success equivalently across cultures. While Bücker et al. (2016) found incongruencies in the concept between a Dutch sample and a Chinese sample, the scale structure was confirmed in an Italian sample (Gozzoli and Gazzaroli, 2018). A further validation study of the German version of the CQS revealed that a bi-factor model with one general cultural intelligence factor, and three subfactors fit German participants best (Greischel et al., 2021).

In contrast to Van Dyne et al. (2008), van der Zee and van Oudenhoven (2000) claim that an individual’s personality is critical for successful interaction between different ethnic groups. They argue that personality traits as context-unspecific predispositions to act in a certain way should either facilitate or hinder intercultural interactions and predict intercultural success in a generalizable way. They developed the multicultural personality questionnaire (MPQ), which consists of the factors cultural empathy, openness, flexibility, emotional stability, and orientation to action and is often considered as a version of the Neo-Five-Factor-Inventory (Big-5; global personality) that focusses on intercultural interaction. The original long-form contained 91 items, the short form 40 items. However, the scale’s factor structure differed between countries (van der Zee et al., 2004; van der Zee and Brinkmann, 2004; Bene, 2018). In an attempt to overcome these difficulties, factor structure and composition were examined in a Spanish and a Hungarian sample, confirming the initial five-factor structure but deleting items due to particularities regarding culture or language (Summerfield et al., 2021).

In summary, measures of attitudes toward and ability to interact with ethnic and cultural outgroups either have not been tested for their cultural comparability, or the results indicate fundamental differences. Although existing models claim for themselves to be generalizable across cultures, large-scale cross-cultural scale validation studies, or culture-specific scale evaluations are scarce. This is considered critical, as linguistic and cultural idiosyncrasies (i.e., in relation to relations of cultural subgroups, support of immigration and integration policies and attitudes like xenophobia) may lead to cultural differences regarding understanding of and emotional connotations toward certain expressions. To investigate such differences and contribute to the understanding of cross-cultural differences and commonalities, we chose three countries with remarkably different history and current situation regarding immigration to compare to a western country, Germany.

Referring to Hofstede Insights (2022), Hungary is considered a strongly individualistic culture. Immigration and emigration have been a reality since the birth of the country, given its geopolitical location. At times, Hungary has been quite a multicultural society (Gozdziak, 2019). Today’s growth of national pride appears to be generated by the economic and political structures. Since 1989, strong ethnocentrism and xenophobia have characterized public attitudes and the political ideologies justifying national existence are important determinants of national identity. Economic nationalism is manifested in the protection of Hungarian goods; political nationalism emphasizes the political supremacy of national interests; and cultural nationalism draws a sharp line between perceived Hungarian and alien cultures, giving unilateral political preference to the former (Örkeny, 2005; Gozdziak, 2019).

According to the Czech Population and Housing Census (2014), the Czech Republic is characterized by national homogeneity dominated by Czech nationality. Czech culture is considered to be rather individualistic (Hofstede Insights, 2022). While Czech politics are not predominantly characterized by xenophobia, the idea of a shared national past and a lost golden age plays an important role in Czech identity formation (Kolaříková, 2021). Thereby, Chlup (2020) emphasizes the antagonism between a pro-Western (pro EU, pro-immigration) perspective and particularistic tendencies that might be rooted in disillusion after post-revolutionary enthusiasm, which is important for understanding Czechs attitudes toward immigration.

Serbia is a strongly collectivist country with a long tradition of emigration, a specific economic and political emigration context, a large number of its citizens abroad, as well as their very heterogeneous geographic distribution and differentiated structures (Predojevic-Despic and Penev, 2016; Hofstede Insights, 2022). In this context, Serbian national identity is manifested, with awareness of history and tradition, medieval heritage, and cultural unity, despite Serbs living under different empires. Three elements, together with the legacy of the Nemanjić dynasty, were crucial in forging identity and preservation during foreign domination: the Serbian Orthodox Church, Kosovo Myth, and the Serbian language (Trbovich, 2009).

Germany is an individualistic country with over one fourth of population having a migration background (Hofstede Insights, 2022). However, political consensus until the first half of the 2000s was that Germany is not and shall not be an immigration country. Political and individual positions still refer to this perspective frequently (Brinkmann and Sauer, 2016). As a result, there are assimilationist and xenophobic tendencies in the German population (KONID Survey, 2019).

## Methodology

### Research question and hypotheses

The central research question of this study focusses on identifying cross-cultural and comparable (etic perspective) as well as culture-specific (emic perspective) aspects of intercultural relations in the four investigated countries. In order to contribute to the understanding of ethnic/cultural intergroup attitudes and competencies in Eastern Europe compared to a Western European country (Germany), we explored commonalities and differences in structures, difficulty, sensitivity, and factor loadings of established measures for ethnic identity, prejudice, acculturation strategies, cultural intelligence, and multicultural personality, putting a special focus on potential idiosyncratic differences (also see van de Vijver and Leung, 1998). Thereby, we expected the factor structures to differ between samples, except for cultural intelligence, which has been found to be relatively stable across cultures (Gozzoli and Gazzaroli, 2018; Greischel et al., 2021). We also expected a two-factor solution differentiating blatant and subtle prejudices in line with the original scale. Single prejudice expressions are expected to be connotated culture-specifically due to linguistic idiosyncrasies of what is considered blatant or subtle. The same aspects may differ between cultural groups in whether being positively or negatively associated, blatant, or subtle (Pettigrew and Meertens, 1995; Genkova et al., 2021). However, due to sensitization in the course of public discourse, it was expected that more items of the prejudices scale would be related more strongly to blatant prejudice compared to the original scale from 1995. Culture-specific perspectives toward intergroup relations would also be found in both, item sensitivities around zero and critically high or low item difficulties differing between samples. This leads to the expectation that in each cultural sample, different items show item sensitivities lower than 40, together with item difficulties lower than 20 or higher than 80. Table 1 summarizes the hypotheses:

**Table 1:** Hypotheses.

H1	Ethnic identity
H1a	Different factor structures between samples
H1b	Different factor loadings between samples
H1c	Different item sensitivities lower than 40, difficulties exceeding 20 or 80
H2	Subtle and Blatant Prejudices
H2a	Different factor structures between samples
H2b	Two-factor solution for all samples
H2c	Different factor loadings between samples
H2d	More items relating to the blatant scale
H2e	Different items sensitivities lower than 40, difficulties exceeding 20 or 80
H3	Acculturation strategies
H3a	Different factor structures between samples
H3b	Different factor loadings between samples
H3c	Different items sensitivities lower than 40, difficulties exceeding 20 or 80
H4	Cultural intelligence
H4a	Four-factor solution in all samples
H4b	Factor structure according to the original scale
H4c	Different factor loadings between samples
H4d	Different items sensitivities lower than 40, difficulties exceeding 20 or 80
H5	Multicultural personality
H5a	Different factor structures between samples
H5b	Different factor loadings between samples
H5c	Different items sensitivities lower than 40, difficulties exceeding 20 or 80

### Sample and procedure

We conducted a secondary data analysis on cross-sectional data collected by the study of Genkova (2021). In the primary analysis, Genkova et al. (2021) conducted confirmatory factor analyses and calculated item reliability and invariances in order to analyze relationships between intercultural competence and prejudices across cultures. This study goes beyond this approach by focusing quantitatively and qualitatively solely on critical items in the respective scales in all samples. The data was collected by convenience sampling and the survey platform LimeSurvey. Participants could access the study *via* desktop computers and mobile devices. Participation took around 20 min on average. All questionnaires were presented in the respective language of the sample. Scales were translated with forward-backward translation by native speakers in the two respective languages (e.g., English – Czech). Prior to participation, participants were asked for their informed consent (data processing for scientific purposes, skipping the study without consequences for them at any time). As the scope of the study is partially explorative, hypotheses were not preregistered.

The data set consisted of a total of 1,027 academic participants without an immigrant background from Germany (421 participants), Czech Republic (223 participants), Serbia (209 participants), and Hungary (174 participants). The German participants belonged to two samples, which were evaluated separately in the work of Genkova et al. (2021). In this study, the German samples were merged into one group, as no major differences regarding demographical background were given. Participants in the overall sample were between 16 and 71 years old, with an average of 25.58 years. The majority were female (70.01%), 27.17% were male, and 29 participants gave no answer. Twenty-four participants had an educational degree comparable to junior high school, 481 had a high-school diploma, 447 were holding a university degree, and 34 had a PhD. The samples included a higher percentage of women than the average student population in the investigated countries. Moreover, the German and Hungarian samples were older on average than the Serbian and Czech samples which might refer to the higher average entrance and exit age for higher education and should be kept in mind while interpreting the results. An overview of demographical data for all samples is available in supplemental material, Supplementary Table A1.

### Scales

The following section describes operationalization of each variable as well as summaries of calculations of internal consistency. Cronbach’s alpha for all scales in each sample is applicable in Supplementary Appendix Table A2.

#### Ethnic identity

To test identification with and commitment to ethnic in- and outgroups, the MEIM was included in the Questionnaire. This scale was developed by Phinney (1992) in an American context. The scale was designed to assess orientation toward one’s ingroup with 14 items. Additionally, Phinney (1992) assessed an opposing factor of orientation towards the outgroup with six items. Other studies found inconsistent results on whether ingroup orientation should be divided into one or two factors, exploration and commitment (e.g., Roberts et al., 1999; Yancey et al., 2003; Ganeva and Rasticova, 2013; Schachner et al., 2016). In this study, Cronbach’s alpha for the whole scale was between 0.681 and 0.801, for the subscale attitudes towards the Ingroup, Cronbach’s alpha was between 0.733 and 0.820, and for the subscale attitudes toward the outgroup, Cronbach’s alpha was between 0.682 and 0.780. Thus, the scale and its subscales showed acceptable internal consistencies. Items are, for example “I try not to join in activities with people from other people groups” or “I am glad to belong to my people group”.

#### Ethnic prejudice

Ethnic prejudice was measured with the BSP, developed by the American and Dutch researchers Pettigrew and Meertens (1995), who provided evidence for the scales validity among European countries and immigrants from non-European countries. Threat/rejection and intimacy are the aspects of blatant prejudice. Six items constituted the threat/rejection subscale, and the intimacy subscale had four items. The subtle prejudice subscale divides into the dimensions traditional values (four items), cultural difference (four items), and positive emotions (two items). In the Serbian sample, only five items of the threat/rejection subscale were applied as other items were considered as especially critical and would potentially have comprised data collection. As the assessment would naturally not be comparable, the items for ethnic prejudice from the Serbian sample were not included in further investigations. Cronbach’s alpha was >0.640 for the separate subscales, and > 0.724 for the scale as a whole. According to Genkova et al. (2021), the scale structure was confirmed for the German samples, but not for the Hungarian and Czech samples. Items included “Refugees have jobs that the [nationals] should have” and “Refugees living here teach their children values and skills different from those required to be successful in [country]”.

#### Acculturation strategy

To measure the preferred acculturation strategy, the 13-item-scale by van Dick et al. (1997) was used in the questionnaire. This scale was applied to a German sample, while the preceding research regarding acculturation strategies was mostly conducted in Canadian samples (Berry, 1992). In the scale of van Dick et al. (1997), five items aimed to measure integration, five items to measure assimilation, and three items to assess separation. However, in the original study, a single-factor solution was found best fitting for the scale, having integration at one end of the dimension, and assimilation/separation on the other end. Reliability for the whole scale was hardly acceptable to unacceptable, as Cronbach’s alpha was found between 0.611 and 0.789, except for the Serbian sample, where Cronbach’s alpha was 0.118, indicating that there was little internal consistency in this sample. Items are, for example “It would be great, when all ethnic groups keep their own culture in [country]” and “Teachers should promote the contact between students of different ethnical backgrounds”.

#### Intercultural intelligence

Intercultural intelligence was assessed with the 20-item-form of the CQS by American researcher Van Dyne et al. (2008). The scale is divided into four dimensions: metacognitive (four items), cognitive (six items), motivational, and behavioral (both five items). Reliability values were acceptable for all samples. Genkova et al. (2021) found that configural measurement invariance was not given, and the scale structure was confirmed only for the Czech sample and nearly confirmed for the German sample. Items include “I know the legal and economic systems of other cultures” and “I enjoy interacting with people from different cultures”.

#### Multicultural personality

This construct was measured with the 40-item-version of the MPQ by Dutch researchers van der Zee and van Oudenhoven (2000), who developed the scale in a Dutch sample. Each of the five dimensions had eight items. Explorative factor analyses suggested different factor compositions and numbers for each subsample. Reliabilities were calculated with Cronbach’s alpha, which ranged from 0.675 to 0.816 in the subsamples. Configural measurement invariance was found very low, and the construct structure was not confirmed in any of the samples. Example items are “Works according to strict scheme” and “Makes contacts easily”.

## Results

The following section describes the results of the study. Calculations included explorative factor analyses (principal factor analysis with varimax rotation) for each sample to calculate factor loadings using SPSS. Numbers of factors were chosen as suggest by the scree-test. The Bartlett test was turned out significant in all cases (*p* < 0.001), while the Kaiser–Meyer–Olkin criteria was significant in none of the cases (*p* between 0.737 and 0.909). We further calculated item difficulty and sensitivity for each sample with the open source version of RStudio. Items were classified as critical if single items were found among different factors than intended in the original scale composition, or if item difficulty was lower than 20 or higher than 80, or if item sensitivity was lower than 0.400. The emerging factors and potentially critical items were then interpreted qualitatively regarding implications for culturally different connotations and understandings and analyzed using cultural and historical background information. While we report the main findings in this text, factor score weights for all samples are available in Supplementary Appendix Table A3. Item difficulties and item sensitivities are provided in Supplementary Appendix Table A4.

### Ethnic identity

*H1a*: Different factor structures between samples.

*H1b*: Different factor loadings between samples.

In the explorative factor analysis, one factor emerged for attitudes toward the ingroup and one for attitudes towards the outgroup in the Hungarian and Serbian samples. This contradicted H1a. The Czech sample did not produce a positive definite correlation matrix. In the German sample, the ethnic identity items were attributed to two distinct factors. One factor included the items “I feel a strong attachment towards my own ethnic group” “I have a lot of pride in my ethnic group and its accomplishments” “I have a strong sense of belonging to my own ethnic group” “I am happy that I am a member of the group I belong to” “I feel good about my cultural or ethnic background” and “I participate in cultural practices of my own group, such as special food, music, or customs” The Items: “In order to learn more about my ethnic background, I have often talked to other people about my ethnic group” “I have a clear sense of my ethnic background and what it means for me” “I have spent time trying to find out more about my own ethnic group, such as its history, traditions, and customs” “I think a lot about how my life will be affected by my ethnic group membership” “I really have not spent much time trying to learn more about the culture and history of my ethnic group” “I understand pretty well that my ethnic group membership means to me, in terms of how to relate to my own group and other groups” “I am not very clear about the role of my ethnicity in my life” loaded on the other factor. In the Hungarian sample, the originally ingroup item “I think a lot about how my life will be affected by my ethnic group membership” showed stronger association with the attitudes toward outgroups, as factor loadings were higher on the second factor, but similarly low on both factors (Factor 1: 0.147; Factor 2: 0.178). Accordingly, this item had stronger sensitivity in the outgroup factor (*s* = 0.335) than in the ingroup subscale (*s* = 0.187). Furthermore, the factor loadings were consistent with the scale conception in all samples (H1b).

*H1c*: Different items sensitivities lower than 40, difficulties exceeding 20 or 80.

Most of the MEIM items showed uncritical item sensitivities (s larger than 0.400) and item difficulties (*d* between 30.62 and 78.16) in all samples. However, various items showed critical values in the respective cultural samples in line with H1c. In the Hungarian sample, item sensitivity was critical for the item “I am not very clear about the role of my ethnicity in my life” for both factors, attitudes toward the ingroup (*s* = 0.122) and attitudes toward the outgroup (*s* = −0.152), yet showed no critical item difficulty. The item “I really have not spent much time trying to learn more about the culture and history of my ethnic group” was negatively associated with the scale (*s* = −0.921). Lowest sensitivities were found among the reversed items. In the Serbian sample, the item “I do not try to become friends with people from other ethnic groups” had critical sensitivity (*s* = 91.47). In this sample, the reversed items showed the highest sensitivities. In the German sample, two items from the outgroup attitudes subscale showed difficulties larger than 0.8. Further, the item “I am active in organizations or social groups that include mostly members of my own ethnic group” had critically low sensitivity (*s* = 0.272).

### Blatant and subtle prejudice

*H2a*: Two-factor solution for all samples.

*H2b*: Two-factor solution for all samples.

*H2c*: Different factor loadings between samples.

*H2d*: More items relating to the blatant scale.

The Serbian sample was excluded from factor analyses, as only five threat items were included in the Serbian questionnaire. We found different factor structures in all of the samples in line with H1a. Accordingly, the composition of factors varied between the samples (H1b). As no blatant vs. subtle prejudice factor solution emerged across all samples, H2d could not be tested. No items showed factor loadings contrary to the intended meaning of the item in the original scale (H2c).

In the Hungarian sample, the item “How different or similar do you think refugees living here are to other Hungarian people like yourself – in how honest they are” was ascribed to the factor cultural differences, while originally belonging to the threat/rejection subscale, as the factor loadings suggested stronger association with the factor cultural differences (0.512) than with the threat factor (0.316). The item “Most politicians in Hungary care too much about refugees and not enough about the average Hungarian person” from the threat subscale was linked to the factor with items mostly from the traditional values subscale. Apart from that, the original factor structure was confirmed for the Hungarian sample. In the Czech sample, a three-factor solution emerged. The first factor combined the dimensions threat/rejection and traditional values. Another factor combined the subscales cultural differences and positive emotions, including the originally threat/rejection item “How different or similar do you think refugees living here are to other Czech people like yourself-in how honest they are”. In a five-factor solution, this item was affiliated with the positive emotion items. Finally, one factor emerged containing all intimacy items, as well as the traditional values item “I would be willing to have sexual relationships with a refugee” In the German sample, a two-factor solution was suggested by the Scree-plot. One factor represented the blatant prejudice subscale, while the traditional values items “Refugees living here should not push themselves where they are not wanted” and “It is just a matter of some people not trying hard enough” were also affiliated with this factor. Intimacy item “I would be willing to have sexual relationships with a refugee” was found to rather relate to the subtle prejudice items. Positive emotion items scored similarly high on both factors.

No differences regarding the item polarity were found in any of the samples.

*H2e*: Different items sensitivities lower than 40, difficulties exceeding 20 or 80.

Various items showed item sensitivities below 0.4, especially in the Hungarian sample. The lowest item sensitivities were found for the item “Suppose that a child of yours had children with a person of very different color and physical characteristics than your own. Do you think you would be bothered if your grandchildren did not physically resemble the people on your side of the family?” (*s* = 0.000) in the Hungarian sample, and “In their religious beliefs and practices” (*s* = 0.066) in the German sample.

Item difficulties were uncritical for most items, reaching from 22.16 to 87.94. In the Hungarian sample, the lowest item difficulties were found for the cultural differences subscale, ranging from 12.30 to 34.22. The same was found in the Czech sample (*d* between 14.85 and 31.88). In the German sample, two items had critically low item difficulties, namely “I would not mind if a suitably qualified refugee person was appointed as my boss” (*d* = 11.41) and “I would not mind if a refugee person who had a similar economic background as mine joined my close family by marriage” (*d* = 14.19). Four blatant prejudice items had difficulties between 0.8 and 0.9, in line with H2e.

### Acculturation strategy

*H3a*: Different factor structures between samples.

*H3b*: Different factor loadings between samples.

the original scale structure was confirmed, supporting H3a. Table 2 shows the factor structure in the different subsamples. While the Hungarian, Czech, and German sample showed similarities in their factor composition, the emerged factor structure of the Serbian sample differed from the other samples. In all samples, the first factor included items from the original separation and assimilation scale. The item “Teachers should promote the contact between students of different ethnical backgrounds” showed high factor loadings on all factors throughout the different samples. In the Hungarian sample, one factor with three integration items compiled. Similarly, the Czech sample showed one factor with all items originally affiliated with integration, except for the item “If many different ethnic groups exist in Hungary, it will be difficult to solve problems” In the Serbian sample, the first factor included all three separation items, of which two scored negatively on the first factor, while the item “Members of different ethnic groups should live separated in all areas of life, to avoid problems between the groups” showed positive factor loadings giving support to H3b. Likewise, assimilation item “Migrants should not show their foreign-cultural habits in the public” had a negative factor loading on factor one, while the other two assimilation items were positively associated with this factor. In the German sample, the items did not assemble according to the original scale. One factor consists of a variety of items from all original subscales, another factor includes three items from the original assimilation subscale, and one factor consists of two items, originally belonging to the integration subscale.

*H3c*: Different items sensitivities lower than 40, difficulties exceeding 20 or 80.

**Table 2:** Factor structure of the acculturation scale compared between the subsamples.

Factor	Hungary	Czech rep.	Serbia	Germany
Factor 1	Sep 2	Sep 2	Sep 2 (−)	Sep 2
	Ass 2	Ass 2	Ass 2	Ass 2
		Sep 3	Sep 3	Sep 3
	Ass 3	Ass 3	Ass 3	
	Ass 4		Ass 4 (−)	Ass 4
	Int 5 (−)			Int 5 (−)
			Int 2	Sep 1
			Int 4	Int 3 (−)
			Sep 1 (−)	
Factor 2	Ass 5	Ass 5	Ass 5	Ass 5
	Int 4	Int 4		Int 4
	Ass 1	Ass 1		Ass 1
	Sep 1	Sep 1	Int 5	Ass 3
		Ass 4	Int 1	
Factor 3	Int 2	Int 2		Int 2
	Int 1	Int 1		Int 1
	Int 3	Int 3	Int 3	
		Int 5	Ass 1	

(−) negative loading on the affiliated factor.

Against H3c, item difficulties were acceptable throughout the scales and samples, ranging from 18.87 to 83.17. Item sensitivities were acceptable and larger than 0.302 in most cases. In the Hungarian sample, the item “People, who come to Hungary, should adapt their behavior to the Hungarian culture” had critical sensitivity (*s* = 0.106). In the Czech sample, the integration items “It would be great when all ethnic groups keep their own culture in the Czech Republic” (*s* = 0.282) and “If many different ethnic groups exist in the Czech Republic, it will be difficult to solve problems” (*s* = 0.061) had unsatisfactory sensitivities, supporting H3c. In the Serbian sample, the item sensitivities align with the factor structure, as item sensitivities were found negative for “If many different ethnic groups exist in Serbia, it will be difficult to solve problems” (*s* = −1.464), “People, who come to Serbia, should adapt their behavior to the Serbian culture” (*s* = −0.053), “Migrants should not show their foreign-cultural habits in the public” (*s* = −0.786), and “Members of different ethnic groups should live separated in all areas of life, to avoid problems between the groups” (−1.178).

### Intercultural competence

*H4a*: Four-factor solution in all samples.

*H4b*: Factor structure according to the original scale.

*H4c*: Different factor loadings between samples.

*H4d*: More items relating to the blatant scale.

The original factor structure of the CQS was confirmed throughout all subsamples, supporting H4a and b. Factor loadings were consistent throughout all items and samples, which contradicts H4c. All items showed item sensitivities higher than 0.300 against the assumption of H4d, and all item difficulties were in an acceptable range, between 20 and 80, thus contradicting H4d. The only item with critical difficulty and sensitivity was “I know the rules (vocabulary, grammar) of other languages” in the Czech sample, where it showed a high difficulty (*d* = 87.12) and low sensitivity (0.242).

### Multicultural personality

*H5a*: Different factor structures between samples.

*H5b*: Different factor loadings between samples.

While the factor structure partially differed in the samples (H5a), the items did not load contrary to the original scale (H5b; items that load negatively on contradicting factors are not seen as loading against expectations). Table 3 displays the factor structure in the four samples.

**Table 3:** Factor structure of the multicultural personality questionnaire compared between the subsamples.

Factor	Hungary	Czech rep.	Serbia	Germany
Factor 1	Emp1	Emp1	Emp1	Emp1
	Emp2	Emp2	Emp2	Emp2
	Emp3	Emp3	Emp3	Emp3
	Emp4	Emp4	Emp4	Emp4
	Emp5	Emp5	Emp5	Emp5
	Emp6	Emp6	Emp6	Emp6
	Emp7	Emp7	Emp7	Emp7
	Emp8	Emp8	Ope4	Emp8
	Ope4	Stabi8	Ope7	Ope6
	Ope7		Ope8	
	Ope8			
Factor 2	Flexib1	Flexib1	Flexib1	Flexib1
	Flexib2	Flexib2	Flexib2	Flexib2
	Flexib3	Flexib3	Flexib3	Flexib3
	Flexib4	Flexib4	Flexib4	Flexib4
	Flexib5	Flexib5	Flexib5	Flexib5
	Flexib6	Flexib6	Flexib6	Flexib6
	Flexib7	Flexib7	Flexib7	Flexib7
	Flexib8	Flexib8	Flexib8	Flexib8
				Ope5-
Factor 3	Ope1	Ope1	Ope1	Ope1
	Ope2	Ope2	Ope2	Ope2
	Ope3	Ope3	Ope3	Ope3
	Ope5	Ope4	Stabi5	Ope4
	Ope6	Ope5	Stabi8	Ope7
	Stabi8	Ope6		Ope8
		Ope7		
		Ope8		
Factor 4	Orient1	Orient1	Orient1	Orient1
	Orient2	Orient2	Orient2	Orient2
	Orient3	Orient3	Orient3	Orient3
	Orient4	Orient4	Orient4	Orient4
	Orient5	Orient5	Orient5	Orient5
	Orient6	Orient6	Orient6	Orient6
	Orient7	Orient7	Orient7	Orient7
	Orient8	Orient8	Orient8	Orient8
		Stabi6		
Factor 5	Stabi1	Stabi1	Stabi1	Stabi1
	Stabi2	Stabi2	Stabi2	Stabi2
	Stabi3	Stabi3	Stabi3	Stabi3
	Stabi4	Stabi4	Stabi4	Stabi4
	Stabi6	Stabi5	Stabi6	Stabi5
	Stabi7	Stabi7	Stabi7	Stabi6
				Stabi7
				Stabi8

(−) negative loading on the affiliated factor.

In the Hungarian sample, the scales flexibility and orientation to action emerged as intended in the original composition. Cultural empathy formed a factor, including the items “Has broad range of interests” “Seeks people from different backgrounds” and “Likes to imagine solutions to problems” Those three items originally belonged to the openness scale, and scored high on both subscales, openness and cultural empathy. The item “Keeps calm when things do not go well” was originally set in the subscale emotional stability, but scored higher on the openness factor (0.509). Loading directions were consistent throughout.

In the Czech sample, the factors flexibility and orientation to action were confirmed by the factor analysis. Empathy formed a single factor, including the item “Is not easily hurt” which originally belonged to the scale emotional stability. The item “Is insecure” was found among the orientation to action scale, while originally belonging to the emotional stability scale. Loading direction was consistent throughout.

In the Serbian sample, no factor emerged according to the original scale structure. Some of the empathy items formed one factor, including the items “Has broad range of interests” “Seeks people from different backgrounds” and “Likes to imagine solutions to problem”, all originally from the openness scale. Flexibility constituted one factor, together with the item “Has feeling for what’s appropriate in culture” which loaded negatively on this scale (−0.323), while also showing relatively high affiliation with the openness scale (0.298). Orientation to action formed one factor, together with the item “Sets others at ease” which was originally ascribed to the empathy scale. Emotional stability emerged as a factor without the items ascribed to different factors. Furthermore, openness was found as a factor, including the items “Keeps calm when things do not go well” and “Is not easily hurt” which were initially set in the subscale emotional stability.

In the German sample, the factor structure was almost confirmed entirely, except from openness items “Is a trendsetter in societal developments” which was attributed to the flexibility subscale, and the item “Has feeling for what’s appropriate in culture” which was affiliated with cultural empathy.

*H5c*: Different items sensitivities lower than 40, difficulties exceeding 20 or 80.

In the Hungarian sample, item sensitivities for the overall scale were almost acceptable, lying between 0.259 and 0.635. The items of the subscale flexibility showed sensitivities below 0.208, except for the item “likes routine” (*s* = 0.371). In the Czech sample, the item “Works according to rules” showed a negative item sensitivity (*s* = −0.008). All other item sensitivities laid between 0.056 and 0.586, thus indicating poor overall item-scale-correlations. Openness items were all acceptable, between 0.373 and 0.490, and items concerning contact with others had the highest item sensitivities, between 0.500 and 0.586. In the Serbian sample, regarding item sensitivity, the dimensions cultural empathy, flexibility, and openness had acceptable sensitivities ranging from 0.308 to 0.678, except for the openness item “Starts a new life easily” (*s* = 0.267) and the flexibility item “Functions best in a familiar setting” (*s* = 0.120). Orientation to action and emotional stability items indicated poor item-scale-correlations, as sensitivities ranged from 0.032 to 0.367, supporting H5c. No item had negative sensitivity. In the German sample, item sensitivities were acceptable, larger than 0.409, with some exceptions. The subscale social initiative showed the least sensitivities, with three items with sensitivities close to zero, and the item “is a trendsetter” had critical sensitivity (*s* = 0.220). Against H5c, all item difficulties were in an acceptable range, between 32.27 and 88.81. Difficulties >80 were found almost exclusively for items of the cultural empathy subscale in all samples. For example, among Hungarian participants, six cultural empathy items showed difficulties >80.

## Discussion

The results show both culture-specific and cross-cultural tendencies for the investigated scales. Established constructs that are considered comparable (ethnic identity, prejudice, acculturation orientation, intercultural intelligence, multicultural personality) show limitations of comparability regarding factor structure, difficulty, and sensitivity. Even though results might be biased through the small sample (some of them also contain more women with higher education), linguistic and cultural connotations appear to be related to different understandings of constructs which should be considered in tighter or looser culture-specific conceptualizations in the future.

### Ethnic identity

Contradicting H1a, we found one factor for orientation toward the ingroup and one for orientation toward the outgroup in the Hungarian and Serbian samples. In the Czech sample the covariance matrix did not compile while we found three factors in the German sample: one factor outgroup orientation, two factors similar to confirmation and belonging as described by Roberts et al. (1999). The one-factor solution for ethnic identity (theoretically comprising the subdimensions belonging, development and practices) stands in contrast to more recent results (Roberts et al., 1999; Yancey et al., 2003; Navarro et al., 2020) and theoretical considerations that emphasize the multidimensional character of identity (Ashmore et al., 2004). The factor loadings were consistent with the scale conception in all samples, indicating that all of the MEIM items were understood and associated the same way in all samples, thereby falsifying the assumption that factor loadings differed due to linguistic and cultural idiosyncrasies (H1b). The items that differed in factor affiliation or item characteristics show that minor differences in the understanding and connotation of ethnic identity exist. This may be due to cultural and historic developments of ethnic identity in each culture, as for example, pride about one’s ethnic identity may be interpreted as positive (e.g., patriotism in the USA or pride in Hungary) or negative (e.g., nationalism in Germany).

In the Hungarian sample, two items were unrelated to the other items, both of which asked about pride in and attachment to the ethnic ingroup. This could be explained by a high average level of nationalism/national pride in Hungary, as also reported by Weiss (2003). It is also possible that the results are caused by the comparably higher age of Hungarian participants, which could lead to a different importance and connotation of nationalism/national pride (Phinney and Ong, 2007). The high amount of critical item sensitivities among German participants may be explained by different understandings of Germany as either an ethnic identity, or supra-ethnic identity comprised of many ethnic groups living together within the German national borders (Fischer and Mohrman, 2021). Among Serbian participants, three items of the MEIM were independent of the total scale, while having acceptable item difficulties. These items assessed how content people are with their ethnic identity, which maybe shows that for the Serbian participants, being content with the ethnic identity constitutes a basic attitude that is not affected by other attitudes. High item difficulties were found among items that assessed intergroup contact, possibly due to the importance of intercultural friendships in the Serbian post-conflict society (Žeželj et al., 2017). Future studies should investigate whether this result is generalizable to more representative Serbian samples.

Many samples showed critical sensitivities for the item “I am not very clear about the role of my ethnicity in my life” We argue that this is due to difficulties in understanding the term role in the context of ethnic identity. This result is supported by the findings of Ponterotto et al. (2003), who found the lowest factor loadings in the factor attitudes toward the ingroup for this item as well. As we did not display other potentially relevant aspects of ethnic identity, the results might indicate that multidimensionality of identity works fundamentally differently in the different samples. Even considering heterogeneity across samples, this could mean that some aspects of ethnic identity (in this case the MEIM aspects) are best represented by one factor in some cultures, while members of other cultures might experience belonging and practices quite differently in terms of aspects of values and habits such as national pride or collectivism, for example. Future studies should focus on this topic by considering different forms of social identity formation and cognitive representation of intergroup relations, as suggested by Yuki (2003) and Hamamura (2017), and by applying more sophisticated statistical analyses, such as multigroup analysis in structural equation modeling to entangle cross-cultural differences and commonalities.

### Prejudice

Regarding blatant and subtle prejudice, different factor structures were found for the samples and some items were affiliated differently from the original scale conceptualization. This confirmed H2a. In the German sample, a two-factor solution proved best fitting. Blatant and subtle prejudice were confirmed almost as originally intended, except from two items. The items “If refugees would only try harder, they could be as well off as German people” and “Refugees living here should not push themselves where they are not wanted” were assigned to the blatant prejudice subscale. The intimacy item “I would be willing to have a sexual relationship with a refugee” loaded higher on the subtle prejudice scale, indicating that keeping social distance to refugees was assumed to be more socially accepted compared to the original sample. These results fit the observations of Hofmann (2016) that during the refugee crisis, members of the German put greater emphasis on distance and hierarchical differences with refugees. In contrast, public discourse focused attention on the weakness of integration policies and laws rather than the performance of refugees. This could explain why this kind of “victim-blaming” appeared to be perceived more as a blatant prejudice.

The other samples showed a four-factor solution (Hungary) or a three-factor solution (Czech Republic), similar to the original scale conceptualization. However, the cultural groups varied regarding the classification of items as blatant or subtle expressions. Factor loadings were consistent in whether being positive or negative across the samples, indicating no linguistic or cultural idiosyncrasies, and thus rejecting H2c. The item “In your opinion: How similar or different do you think refugees living here are to other [national] people like yourself – in how honest they are?” was affiliated with the subtle prejudice factor in the Hungarian, Czech, and German samples. This may be due to formulation similarities with the items of the subscale cultural differences. Another explanation for the Czech sample may be a culture-specific association of honesty with positive emotions as described by Dvorakova et al. (2013). In the Hungarian sample, two threat items were assigned to the subfactor traditional values. This is in line with reaction of the Hungarian government to the refugee crisis as described by Rokicka (2021), putting a great emphasis on thread toward Hungarian traditions and way of life. This might also be related to the higher average age of the Hungarian sample, being potentially more prone to authoritarian and thread-related attitudes (Raabe and Beelmann, 2011).

In the Czech sample, all items of the blatant prejudice subscale intimacy showed low item sensitivities. This is consistent with the structure that was found in the factor analysis. Both results imply that blatant and subtle prejudice is different in Czech Republic than suggested by Pettigrew and Meertens (1995). The low difficulty values in the subscale cultural differences might indicate that Czech participants desire to distinguish themselves from other ethnic groups. This may be explained by their high amount of consciousness of international importance compared to other European countries, even though their national pride or egoism has been rated as average (Weiss, 2003). Moreover, one might consider that the younger Czech participants could have other blatant prejudices which are not captured in the scale from 1995. As a consequence, capturing the content of prejudices in several countries would require a cautious analysis of collective intergroup relations (e.g., historical events, wars, conflicts) and of aspects that are considered as important in intergroup relations by different groups in one culture.

The German sample showed high difficulty values in the blatant prejudice items. This may be explained by the public debate since the refugee crisis 2015, as many narratives behind ethnic prejudice were openly discussed and found problematic for living together peacefully (Genkova and Groesdonk, 2021). The low difficulties of two intimacy items indicate that Germans consider hierarchies at work regardless of ethnic background. Similar results were found by Wenz and Hoenig (2020), showing that German participants judged others rather based on social class compared to ethnic background.

In total, the results indicate that blatant and subtle prejudice are generalizable across relatively young people from the investigated countries but might not capture all relevant aspects of prejudices in each country. Single aspects that are included in the BSP items appear to be subject to culture-specific social standards (e.g., qualification as legitimization of leadership regardless of ethnic group) or connotations (e.g., inter-ethnic relationships as positive or negative). Cultural idiosyncrasies may be due to linguistic idiosyncrasies or to collective experiences of a cultural group, such as large-scale immigration. Furthermore, it is not clear to what extent older participants from different countries would show different expressions of subtle and blatant prejudice, as expressions and connotation of political correctness might vary more in older samples. Further cross-cultural investigations should thus examine whether there may be stronger intra-country differences in what should and should not be said between age cohorts than between young, modern, and politically correct participants from different cultures.

### Acculturation strategies

In all samples, a three-factor model was found for acculturation strategies. As expected in H3a, the factor structures varied between the samples, possibly revealing differences regarding the structure of and motives behind the attitudes included in the scale. In the Hungarian sample, one factor emerged that included items concerning acculturation at school, and living out one’s culture in public. One factor included three integration items. The third factor consisted of items that were related to assimilation and separation. In the Czech sample, one factor emerged consisting of four integration items. One factor was compiled from two separation items and two assimilation items, which together resemble the marginalization strategy. One more factor was found, pointing at assimilation strategy, as the separation items included in this factor specifically aimed at migrants’ culture renunciation.

The Serbian sample showed a distinct set of factors. One factor consisted of only two items and aimed at unity between different ethnic groups in Serbia, based on speaking the same language and solving problems together. This was in line with what Trbovich (2009) considered as important aspect of Serbian national identity. Another factor included two integration items and one assimilation item, focusing on learning the Serbian language. The items included in the last factor were from all subscales and aimed at harmony between different ethnic groups and to avoid conflict between them. The factor structure may shed light on Serbs’ strife for unity in their country between different ethnic groups, their understanding of Serbian language as a unifying factor, and their mindset that develops strategies to avoid inter-ethnic conflict. This aligns with the findings of Sakač (2019) as well as Ljujic et al. (2012), who reported assimilation pressure among ethnic minorities in Serbia, especially regarding language.

In the German sample, two items constituted one factor regarding the maintenance of immigrants’ heritage culture. Another factor emerged consisting of assimilation items, and one negatively associated integration item. The last factor pointed at separation, including assimilation items concerning heritage culture deferring of immigrants, as well as negatively related integration items. This way, the underlying construct conception was confirmed, however, with different alignment of items.

Some cross-cultural similarities in attitudes were found. For example, “Children of different ethnic groups should go to different ethnic schools according to their culture” and “Members of different ethnic groups should live separated in all areas of life, to avoid problems between the groups” show very high sensitivities and difficulties in all samples, meeting our expectations of H3c. This may imply that in European countries, mixing people, especially students, is seen as important for integration and that these items best reflect attitudes toward integration.

In line with the findings of Piontkowski et al. (2000) measurement of acculturation strategies was found not to be comparable across cultures, as culture-specific acculturation orientations emerged from the factor analyses. This may be explained by the different reactions of societies to the increasing amount of intercultural contact (e.g., during the European refugee crisis 2015, or increasing labor migration due to economic welfare), leading to different collective experiences with intergroup contact. Differences regarding the affiliation of items and item characteristics may root in linguistic or cultural peculiarities, as well as culture-specific connotations of single aspects (e.g., collective experience of successful integration of ethnic outgroups). Furthermore, differences in the samples might be related to different socio-demographic characteristics. Especially highly educated participants (such as overrepresented in the Hungarian and German sample) might consider suspected differences in education level and fluency of language as more important aspects of identification. In each case, the current results contradict the assumption that the reaction to acculturation of host-culture members might be displayed by two dimensions (contact and adaptation) as suggested by Berry (1980). If we consider acculturation strategies more as normative attitudes rather than reactions to increasing diversity, it becomes much clearer that there will be huge differences in understanding and connotation due to cultural values and norms, as well as current politics and public debate (Guimond et al., 2014). The results further lead to the question, which attitudes might be relevant to members of a certain society that could lead to the factor solutions displayed in this study. Future qualitative studies should examine this issue beyond Berry’s theory.

### Intercultural competence

The results show that cultural intelligence, as operationalized in the CQS, applies to Germany, Hungary, Czech Republic, and Serbia, verifying H4a and H4b, and falsifying H4c. H4d was only confirmed by the item “I know the rules (vocabulary, grammar) of other languages” in the Czech sample, which had low item sensitivity and high difficulty, which may be due to the high level of foreign language skills among the Czech population (Kralova and Dolezelova, 2021). However, it is unclear to what extent this result is generalizable to older Czechs who prefer particularistic policies based on disappointing experiences in the curse of Czech opening toward the West (Chlup, 2020). The surprising cultural equivalence of constructs, dimensions, as well as item affiliations and characteristics was forecast by the configural measurement equivalence found by Genkova et al. (2021) in the same data set. This may be due to the conceptualization of cultural intelligence, which adopts fundamental psychological concepts (such as cognitive or motivational) as dimensions relating to intercultural competence. Likewise, the items of the CQS are formulated so that the general aspects of the respective dimensions are covered and related to intercultural contact (e.g., “I know the legal and economic system of other cultures”). Similarities between samples might also be related to the limited variance in the data due to the restriction to a certain age group.

### Multicultural personality

In general, the original dimensions of the MPQ emerged from the factor analyses in all samples, contradicting H5a. Factor loadings were consistent in all samples, thus falsifying H5b. A general factor of personality was not tested, as researchers argued against such a concept (Revelle and Wilt, 2013). The association of openness items with other subscales, especially cultural empathy, as found in many of the included samples, is supported by the results of Petrović and Vučetić (2014). They found a four-factor-solution best fitting for the Serbian 103-item-version of the MPQ within a Serbian sample, where openness items were affiliated with cultural empathy and orientation to action. In the German sample, openness items were not affiliated with emotional stability, which contradicts the findings of Mieg et al. (2012), who found emotional stability and openness to relate to each other. Further, the factor structure was confirmed in the German sample, except from two items. This sample deviated the least from the original scale conception, which had also been developed with student samples in a western-European environment. This may indicate that geographically and culturally similar groups of people share more common conceptions of multicultural personality than cultures that are more distant from each other.

Furthermore, one factor in the German sample included items related to taking initiative, working environment, and problem solving, possibly indicating that labor and work environment are an important part of German culture (Thomas and Simon, 2007). In the MPQ, items that included statements about intercultural contact situations showed highest sensitivities, which aligns with the definition of intercultural competences as skills that facilitate intercultural contact and relationships (Genkova, 2019). Czech participants were found to have low item sensitivities on the openness and orientation to action items. While it is not clear to what extent this result is biased by the sample socio-demographic characteristic, future studies should focus on the culture-specific aspects of open and socially initiative behavior in cross-culture interaction in the Czech Republic. Only in the Serbian sample, the item “Gets upset easily” had a negative sensitivity, which may be due to a different connotation of this expression in the Serbian collectivist culture. Among Hungarians, almost all cultural empathy items had high difficulty scores, which may imply that the self-perception regarding empathy is systematically shaped by cultural stereotypes in Hungary. This was the only item with critical difficulty related to H5c.

Equivalence between samples was found for only some of the personality dimensions. Possibly, the cultural context, in which the scale was developed (Netherlands, Far East) may have contributed to a rather culture-specific structure of personality traits. The results of Genkova et al. (2021) align with the results of this study, as configural measurement invariance was found unsatisfactory. The differences regarding item characteristics and factor affiliations of single items may be explained by culture-specific concepts of personality dimensions, differences in age and educational degree, as well as by societal standards in terms of what personality is perceived as positive or negative (e.g., being flexible). Such could be assessed by Multiple Indicators Multiple Causes (MIMIC) models in future studies. Possible confounding effects of foreign language skills (van der Zee et al., 2004) were not examined in this study either.

### Limitations

To make reliable analyses, a minimum of 200 participants per sample was suggested by the preceding power analysis by Genkova et al. (2021). This number was reached in four of the five samples, and one sample was close to the critical number. Four different nationalities were included in this study which allows cross-cultural comparisons to a certain degree. We identified critical items based on characteristics and interpreted the effects qualitatively. However, the link between quantitative irregularities and the meaning of the constructs leaves room for interpretation. While abnormalities in the scale structure among the different cultures were pointed out, no novel scale structures or implications for questionnaire application and evaluation in the respective countries were given. Considering the low levels of internal consistency in some of the samples, future research should focus in particular on the extent to which these scales are too tight or too lose and whether they may provide evidence for configural and discriminant validity in the investigated countries. Furthermore, this study did not account for the effects of differential item functioning on the item level of the analysis. The results indicate similar directions of item loadings, which might be an indicator that a stronger expression of a measured attribute is indeed connected to a higher test score in each country. However, we encourage future studies to focus on the item level in different countries as well by applying item-response-theory. Socio-demographic attributes of samples might have an impact on the connotation and expression of cultural traits and define respondents’ attitudes. While German, Serbian, and Czech samples roughly reflected student population in the respective countries in relation to age (except the overly high number of PhDs in the Serbian sample; World Population Review, 2022) the Hungarian sample is older on average than the other samples. Ethnic identity and intergroup attitudes have been found to differ between age groups (Phinney and Ong, 2007; Henry and Sears, 2009). More recent literature suggests that differences in the expression of negative intergroup attitudes might be related to the different social contexts rather than being caused by age (Raabe and Beelmann, 2011; Franssen et al., 2013). Differences in the factor structures might thus be caused partially by the sample-specific distribution of age. Moreover, it is not entirely clear how samples are comparable, as the meaning of educational degrees vary and there are more post-doc participants in the Serbian sample. This might limit the external validity of the results for the population in the respective countries. The results should thus be interpreted carefully and supported by literature on the culture-specific meaning of constructs. Considering the challenge to raise appropriate samples in several countries, future studies are encouraged to use more robust techniques to verify the equivalence of tests such as Multigroup factor analysis *via* equational structural models, Factor Mixture Models, or MIMIC.

## Conclusion

This study explored factor structures and item characteristics of scales that were intended to assess attitudes and abilities related to intercultural relations. Explorative factor analyses were conducted for samples from four countries to display limitations to comparability across cultures. Further, culture-specific connotations of item content were pointed out by critical item characteristics, as well as factor loadings. The critical items and found factor structures were interpreted against the cultural background of the respective sample. Most scales showed comparability in structure in Germany and the Eastern European samples, while various items were interpreted differently. The results indicate that there might be underlying cognitive processes, especially in relation to identity, but the connotation of social categories as well as the relationships between them is likely to vary. The same applies to affective and normative attitudes toward intercultural relations (prejudices and acculturation strategies). Therefore, we strongly recommend the continued development of scales to measure constructs relating to intercultural relations. Future research should thus focus on ways to reduce negative intergroup attitudes by analyzing, for example, ideologies underlying individual attitudes as well as culturally shared stereotypes (Park and Judd, 2005; West and Schoenthaler, 2017).

## Data availability statement

The raw data supporting the conclusions of this article will be made available by the authors, without undue reservation.

## Ethics statement

Ethical review and approval were not required for the study on human participants in accordance with the local legislation and institutional requirements. The patients/participants provided their written informed consent to participate in this study.

## Author contributions

PG, MR, JP, KV, CS, AV, and JB contributed to conception and design of the study. PG is responsible for organizing the data. JH conducted statistical analyses and wrote the initial draft of the manuscript. PG and HS edited the draft. All authors contributed to the article and approved the submitted version.

## Conflict of interest

The authors declare that the research was conducted in the absence of any commercial or financial relationships that could be construed as a potential conflict of interest.

## Publisher’s note

All claims expressed in this article are solely those of the authors and do not necessarily represent those of their affiliated organizations, or those of the publisher, the editors and the reviewers. Any product that may be evaluated in this article, or claim that may be made by its manufacturer, is not guaranteed or endorsed by the publisher.
