# Supplementary material for: A comparative study on culture-specific and cross-cultural aspects of intercultural relations in Hungary, Serbia, Czech Republic, and Germany
Source: Front Psychol. 2022 Oct 6;13:886100. doi: 10.3389/fpsyg.2022.886100 (PMC9584916; doi:10.3389/fpsyg.2022.886100)
Supplement: Supplementary file 1 [file Table_1.docx]

# Appendix

**Table A1** *Demographical data for the total sample and each subsample.*

| Total Sample |  |  |  |
| --- | --- | --- | --- |
| N = 1027 |  |  |  |
|  |  |  |  |
| Age |  |  |  |
| M | SD | Min | Max |
| 25.51 | 7.94 | 16 | 71 |
|  |  |  |  |
| Sex |  |  |  |
| Female | Male |  |  |
| 719 | 279 |  |  |
|  |  |  |  |
| Highest educational degree | | |  |
| Junior high school degree | High school diploma | University Degree | PhD |
| 24 | 481 | 447 | 34 |
|  |  |  |  |
| Hungarian Sample | | |  |
| N = 174 |  |  |  |
|  |  |  |  |
| Age |  |  |  |
| M | SD | Min | Max |
| 30.32 | 8.03 | 20 | 53 |
|  |  |  |  |
| Sex |  |  |  |
| Female | Male |  |  |
| 149 | 25 |  |  |
|  |  |  |  |
| Highest educational degree |  |  |  |
| High school diploma | University Degree | PhD |  |
| 16 | 155 | 3 |  |
|  |  |  |  |
| Czech Sample |  |  |  |
| N = 223 |  |  |  |
|  |  |  |  |
| Age |  |  |  |
| M | SD | Min | Max |
| 21.26 | 1.44 | 18 | 25 |
|  |  |  |  |
| Sex |  |  |  |
| Female | Male |  |  |
| 143 | 80 |  |  |
|  |  |  |  |
| Highest educational degree |  |  |  |
| High school diploma | University Degree |  |  |
| 196 | 27 |  |  |
|  |  |  |  |
| Serbian Sample |  |  |  |
| N = 209 |  |  |  |
| Age |  |  |  |
| M | SD | Min | Max |
| 21.68 | 3.14 | 18 | 29 |
|  |  |  |  |
| Sex |  |  |  |
| Female | Male |  |  |
| 147 | 62 |  |  |
|  |  |  |  |
| Highest educational degree |  |  |  |
| High school diploma | University Degree | PhD |  |
| 65 | 116 | 28 |  |
|  |  |  |  |
| German Sample |  |  |  |
| N = 421 |  |  |  |
|  |  |  |  |
| Age |  |  |  |
| M | SD | Min | Max |
| 27.89 | 9.61 | 16 | 71 |
|  |  |  |  |
| Sex |  |  |  |
| Female | Male |  |  |
| 280 | 112 |  |  |
|  |  |  |  |
| Highest educational degree |  |  |  |
| Junior high school degree | High school diploma | University Degree | PhD |
| 24 | 204 | 149 | 3 |

**Notes** N = Number of participants, M = mean, SD = standard deviation, Min = minimum, Max = maximum.

**Table A2** Cronbach’s Alpha for all subscales in all samples

| Scale | Hungary | Czech Republic | Serbia | Germany |
| --- | --- | --- | --- | --- |
| **MEIM** |  |  |  |  |
| Ethnic Identity | .87 | .72 | .91 | .86 |
| Attitudes towards Outgroups | .78 | .74 | .70 | .77 |
| **CQS** |  |  |  |  |
| Metacognitive | .93 | .84 | .88 | .83 |
| Kognitive | .91 | .80 | .87 | .84 |
| Motivational | .91 | .86 | .87 | .89 |
| Behavioral | .93 | .86 | .92 | .85 |
| **MPQ** |  |  |  |  |
| Empathy | .91 | .82 | .84 | .86 |
| Flexibility | .88 | .75 | .89 | .83 |
| Social Initiative | .54 | .15 | .34 | .19 |
| Emotional Stability | .69 | .43 | .65 | .61 |
| Openness | .88 | .79 | .81 | .71 |
| **Acculturation** |  |  |  |  |
| Integration | .39 | .47 | .66 | .26 |
| Assimilation | .69 | .75 | .41 | .74 |
| Separation | .71 | .64 | .73 | .89 |
| **Prejudice** |  |  |  |  |
| Blatant: Threat | .63 | .73 | .65 | .88 |
| Blatant: Intimacy | .80 | .41 |  | .06 |
| Subtle: Traditional values | .77 | .73 |  | .81 |
| Subtle: Emotions | .76 | .72 |  | .76 |
| Subtle: Similarity | .81 | .72 |  | .75 |

**Table A3a** Factor scores of each sample for the cultural intelligence scale

|  | Hungary | | | | Czech Republic | | | |
| --- | --- | --- | --- | --- | --- | --- | --- | --- |
|  | F1 | F2 | F3 | F4 | F1 | F2 | F3 | F4 |
| Metacognitive 1 | .221 | .206 | .205 | .840 | .114 | .126 | .215 | .799 |
| Metacognitive 2 | .233 | .160 | .155 | .832 | .181 | .076 | .099 | .745 |
| Metacognitive 3 | .225 | .172 | .174 | .810 | .234 | .099 | .214 | .642 |
| Metacognitive 4 | .209 | .192 | .307 | .704 | .207 | .228 | .273 | .561 |
| Cognitive 1 | .836 | .176 | .211 | .251 | .034 | .839 | .131 | .102 |
| Cognitive 2 | .787 | .160 | .232 | .116 | .092 | .746 | .210 | .101 |
| Cognitive 3 | .756 | .270 | .235 | .134 | .109 | .700 | .204 | .194 |
| Cognitive 4 | .728 | .155 | .094 | .235 | .252 | .624 | .207 | .146 |
| Cognitive 5 | .728 | .113 | .144 | .163 | .092 | .488 | .147 | .150 |
| Cognitive 6 | .508 | .191 | .273 | .350 | -.030 | .143 | .092 | .360 |
| Motivational 1 | .246 | .140 | .828 | .201 | .208 | .124 | .710 | .157 |
| Motivational 2 | .270 | .116 | .818 | .187 | .221 | .264 | .690 | .111 |
| Motivational 3 | .145 | .320 | .780 | .114 | .110 | .179 | .672 | .216 |
| Motivational 4 | .139 | .316 | .639 | .201 | .063 | .219 | .640 | .222 |
| Motivational 5 | .243 | .192 | .581 | .330 | .129 | .273 | .614 | .349 |
| Behavioral 1 | .141 | .850 | .138 | .158 | .773 | .122 | .173 | .102 |
| Behavioral 2 | .140 | .849 | .166 | .183 | .760 | .042 | .074 | .088 |
| Behavioral 3 | .145 | .833 | .290 | .144 | .754 | .102 | .157 | .261 |
| Behavioral 4 | .396 | .706 | .149 | .191 | .733 | .092 | .053 | .108 |
| Behavioral 5 | .240 | .673 | .304 | .176 | .554 | .242 | .296 | .051 |
|  | Serbia | | | | Germany | | | |
|  | F1 | F2 | F3 | F4 | F1 | F2 | F3 | F4 |
| Metacognitive 1 | .170 | .211 | .054 | .800 | .118 | .123 | .153 | .701 |
| Metacognitive 2 | .091 | .297 | .211 | .779 | .448 | .150 | .164 | .589 |
| Metacognitive 3 | .304 | .181 | .104 | .749 | .169 | .184 | .091 | .807 |
| Metacognitive 4 | -.040 | .257 | .145 | .699 | .316 | .167 | .189 | .589 |
| Cognitive 1 | .189 | .792 | .152 | .151 | .109 | .653 | .073 | .205 |
| Cognitive 2 | .192 | .775 | .264 | .228 | .095 | .566 | .073 | -.005 |
| Cognitive 3 | .088 | .736 | .023 | .207 | .195 | .722 | .164 | .177 |
| Cognitive 4 | .105 | .724 | .184 | .119 | .084 | .764 | .085 | .050 |
| Cognitive 5 | .087 | .589 | .034 | .109 | .102 | .558 | .077 | .150 |
| Cognitive 6 | .106 | .539 | -.024 | .316 | .217 | .662 | .163 | .110 |
| Motivational 1 | .283 | .158 | .782 | .022 | .723 | .192 | .218 | .256 |
| Motivational 2 | .101 | .159 | .727 | .233 | .689 | .215 | .201 | .192 |
| Motivational 3 | .173 | .054 | .710 | .026 | .719 | .200 | .213 | .197 |
| Motivational 4 | .160 | .205 | .700 | .210 | .747 | .166 | .143 | .168 |
| Motivational 5 | .245 | -.023 | .672 | .048 | .668 | .099 | .267 | .114 |
| Behavioral 1 | .892 | .087 | .225 | .102 | .376 | .146 | .579 | .057 |
| Behavioral 2 | .815 | .147 | .141 | .036 | .257 | .152 | .593 | .215 |
| Behavioral 3 | .777 | .170 | .229 | .193 | .237 | .001 | .661 | .112 |
| Behavioral 4 | .723 | .229 | .185 | .057 | .123 | .165 | .809 | .147 |
| Behavioral 5 | .678 | .107 | .338 | .215 | .113 | .200 | .768 | .093 |

**Table A3b** Factor scores of each sample for the blatant and subtle prejudice scale

|  | Hungary | | | | Czech Republic | | | | | |
| --- | --- | --- | --- | --- | --- | --- | --- | --- | --- | --- |
|  | F1 | F2 | F3 | F4 | F1 | F2 | F3 | F4 | F5 | F6 |
| Threat 1 | .009 | -.008 | .131 | .683 | .705 | -.014 | .097 | .075 | .060 | .005 |
| Threat 2 | .139 | .077 | .403 | .415 | .461 | .063 | .412 | .146 | .199 | -.004 |
| Threat 3 | .168 | .185 | .090 | .572 | .759 | .114 | -.022 | .034 | .021 | .130 |
| Threat 4 | .036 | .003 | .256 | -.054 | .580 | .175 | .355 | -.006 | .042 | -.019 |
| Threat 5 | -.083 | .089 | .020 | .594 | .509 | -.012 | .106 | .069 | .130 | .256 |
| Threat 6 | .512 | .084 | .094 | .316 | .098 | .254 | .039 | .204 | .430 | -.045 |
| Intimacy 1 | -.095 | -.235 | -.030 | -.006 | .227 | .344 | -.048 | .018 | .166 | .360 |
| Intimacy 2 | .215 | .577 | .097 | -.042 | -.136 | .643 | -.047 | .079 | .115 | -.004 |
| Intimacy 3 | .174 | .739 | -.039 | .337 | .177 | .766 | -.182 | -.012 | .090 | -.031 |
| Intimacy 4 | .069 | .894 | .067 | .151 | .131 | .863 | -.066 | .042 | .099 | .087 |
| Traditional 1 | .068 | .181 | .471 | .316 | .379 | -.007 | .429 | .068 | .110 | .223 |
| Traditional 2 | .063 | .124 | .763 | -.022 | .007 | -.188 | .730 | .084 | -.020 | -.101 |
| Traditional 3 | .010 | .061 | .698 | .206 | .200 | -.118 | .706 | .032 | .053 | .154 |
| Traditional 4 | .068 | -.026 | .586 | .320 | .200 | -.105 | .434 | .049 | .050 | .504 |
| Positive Emotions 1 | -.363 | -.311 | -.149 | -.146 | -.085 | -.122 | .016 | -.156 | -.783 | -.147 |
| Positive Emotions 2 | -.360 | -.334 | -.259 | -.046 | -.091 | -.060 | -.107 | -.136 | -.665 | -.050 |
| Subtle 1 | .715 | .192 | .185 | .202 | .112 | .132 | .072 | .561 | .104 | .374 |
| Subtle 2 | .777 | .138 | .055 | -.138 | .075 | -.071 | .212 | .729 | .112 | -.082 |
| Subtle 3 | .759 | .169 | .034 | .108 | .181 | .192 | -.056 | .532 | .119 | .286 |
| Subtle 4 | .527 | .132 | -.050 | -.155 | -.039 | .001 | .000 | .614 | .186 | -.083 |
|  | Germany | | | | |  |  |  |  |  |
|  | F1 | F2 | F3 | F4 | F5 |  |  |  |  |  |
| Threat 1 | .784 | .105 | .212 | .014 | .105 |  |  |  |  |  |
| Threat 2 | .565 | .468 | .059 | .054 | .257 |  |  |  |  |  |
| Threat 3 | .744 | .051 | .264 | .069 | .095 |  |  |  |  |  |
| Threat 4 | .659 | .304 | .179 | .213 | .277 |  |  |  |  |  |
| Threat 5 | .809 | .177 | .279 | .096 | .068 |  |  |  |  |  |
| Threat 6 | .316 | .263 | .285 | .319 | .226 |  |  |  |  |  |
| Intimacy 1 | .240 | .192 | .538 | .118 | .264 |  |  |  |  |  |
| Intimacy 2 | .173 | .158 | .335 | .270 | .429 |  |  |  |  |  |
| Intimacy 3 | .278 | .176 | .705 | .050 | .172 |  |  |  |  |  |
| Intimacy 4 | .342 | .104 | .815 | .119 | .160 |  |  |  |  |  |
| Traditional 1 | .243 | .388 | .330 | .159 | .257 |  |  |  |  |  |
| Traditional 2 | .109 | .698 | .049 | .173 | .160 |  |  |  |  |  |
| Traditional 3 | .251 | .746 | .249 | .115 | .161 |  |  |  |  |  |
| Traditional 4 | .133 | .580 | .255 | .413 | .127 |  |  |  |  |  |
| Positive Emotions 1 | -.187 | -.203 | -.265 | -.216 | -.732 |  |  |  |  |  |
| Positive Emotions 2 | -.138 | -.185 | -.152 | -.144 | -.669 |  |  |  |  |  |
| Subtle 1 | .116 | .271 | .171 | .611 | .136 |  |  |  |  |  |
| Subtle 2 | .096 | .097 | .008 | .731 | .155 |  |  |  |  |  |
| Subtle 3 | .076 | .207 | .032 | .636 | .192 |  |  |  |  |  |
| Subtle 4 | -.011 | -.002 | .066 | .516 | .007 |  |  |  |  |  |

**Table A3c** Factor scores of each sample for the multicultural personality scale

|  | Hungary | | | | | Serbia | | | | | |
| --- | --- | --- | --- | --- | --- | --- | --- | --- | --- | --- | --- |
|  | F1 | F2 | F3 | F4 | F5 | F1 | F2 | F3 | F4 | F5 |  |
| Empathy 1 | .788 | .092 | -.142 | -.011 | .061 | .793 | -.177 | .092 | -.083 | .044 |  |
| Empathy 2 | .802 | .071 | -.109 | .048 | .034 | .663 | -.264 | -.036 | .064 | .126 |  |
| Empathy 3 | .554 | .070 | .012 | .032 | -.009 | .563 | -.211 | .014 | -.087 | .149 |  |
| Empathy 4 | .669 | .224 | -.094 | -.076 | .110 | .398 | .008 | .057 | -.039 | -.030 |  |
| Empathy 5 | .741 | .177 | -.230 | -.110 | .067 | .610 | -.113 | .158 | .026 | .079 |  |
| Empathy 6 | .657 | .170 | -.202 | -.064 | .069 | .679 | -.141 | .082 | -.100 | .177 |  |
| Empathy 7 | .811 | .083 | -.134 | -.006 | .074 | .741 | -.229 | .122 | -.194 | -.036 |  |
| Empathy 8 | .686 | .281 | -.096 | .078 | .135 | .297 | -.057 | .687 | .151 | .332 |  |
| Flexibility 1 | .018 | -.186 | .721 | .111 | .012 | -.309 | .712 | -.002 | .039 | -.155 |  |
| Flexibility 2 | -.129 | -.224 | .730 | -.039 | -.026 | -.295 | .791 | .106 | -.076 | .043 |  |
| Flexibility 3 | -.047 | -.142 | .746 | .011 | -.071 | -.327 | .688 | -.012 | .046 | .008 |  |
| Flexibility 4 | -.236 | -.048 | .769 | .113 | .016 | -.173 | .670 | -.030 | .094 | -.196 |  |
| Flexibility 5 | -.016 | .058 | .663 | .180 | .135 | .038 | .736 | .199 | .249 | .082 |  |
| Flexibility 6 | -.200 | .089 | .598 | .195 | -.045 | .009 | .728 | .188 | .188 | .126 |  |
| Flexibility 7 | -.214 | -.017 | .518 | .217 | .141 | -.036 | .471 | .102 | -.218 | .209 |  |
| Flexibility 8 | -.210 | .004 | .602 | .252 | .053 | -.074 | .728 | .053 | .105 | -.014 |  |
| Social In. 1 | .034 | .386 | -.182 | .110 | .428 | -.079 | -.114 | .373 | -.033 | .292 |  |
| Social In. 2 | .020 | -.171 | .259 | .231 | .596 | -.138 | .175 | .463 | .032 | .008 |  |
| Social In. 3 | .214 | -.179 | .153 | .297 | .636 | .002 | .037 | .606 | .362 | .079 |  |
| Social In. 4 | .115 | .411 | -.038 | .036 | .671 | .229 | .029 | .740 | .190 | .279 |  |
| Social In. 5 | .129 | .400 | -.081 | -.037 | .631 | .145 | .103 | .613 | .101 | .016 |  |
| Social In. 6 | .086 | .424 | -.038 | -.042 | .612 | .205 | .126 | .665 | .037 | .132 |  |
| Social In. 7 | .244 | .276 | .013 | .007 | .740 | .231 | -.038 | .775 | .130 | .044 |  |
| Social In. 8 | -.019 | -.180 | .207 | .457 | .583 | .131 | .212 | .531 | .290 | -.284 |  |
| Stability 1 | -.119 | .190 | .225 | .722 | .066 | -.079 | .235 | .056 | .715 | .071 |  |
| Stability 2 | .071 | .183 | .144 | .746 | -.046 | -.143 | .142 | .130 | .747 | .152 |  |
| Stability 3 | -.167 | .129 | .128 | .691 | .172 | -.006 | .026 | .092 | .840 | .055 |  |
| Stability 4 | .053 | -.084 | -.020 | .682 | .037 | -.162 | -.021 | .178 | .586 | .038 |  |
| Stability 5 | .098 | .722 | -.028 | .178 | -.066 | .261 | .073 | -.057 | .311 | .566 |  |
| Stability 6 | -.056 | .089 | .163 | .722 | .244 | -.035 | .013 | .309 | .484 | .280 |  |
| Stability 7 | .007 | -.067 | .252 | .573 | .006 | -.027 | .072 | .188 | .741 | .042 |  |
| Stability 8 | .061 | .509 | -.017 | .210 | -.028 | -.066 | .024 | .056 | .269 | .583 |  |
| Openness 1 | .214 | .688 | -.177 | -.032 | .110 | .385 | .092 | .248 | .063 | .606 |  |
| Openness 2 | .330 | .650 | -.156 | -.048 | .091 | .385 | .053 | .283 | .108 | .570 |  |
| Openness 3 | .267 | .652 | .013 | .074 | .156 | .301 | .053 | .115 | .259 | .412 |  |
| Openness 4 | .535 | .494 | -.064 | -.049 | .158 | .556 | -.054 | .190 | -.020 | .488 |  |
| Openness 5 | .319 | .620 | -.004 | .009 | .324 | -.069 | -.459 | .081 | -.054 | .524 |  |
| Openness 6 | .384 | .517 | -.074 | -.070 | .253 | .291 | -.323 | .127 | -.176 | .298 |  |
| Openness 7 | .384 | .367 | -.066 | -.113 | .234 | .333 | .123 | .312 | -.316 | .241 |  |
| Openness 8 | .540 | .353 | -.073 | -.087 | .155 | .468 | -.002 | .258 | -.258 | .419 |  |
|  | Serbia | | | | | | Germany | | | | |
|  | F1 | F2 | F3 | F4 | F5 | F6 | F1 | F2 | F3 | F4 | F5 |
| Empathy 1 | -.188 | .761 | .125 | -.111 | .142 | -.096 | .756 | -.002 | -.062 | .025 | -.075 |
| Empathy 2 | -.267 | .654 | -.027 | .056 | .169 | -.029 | .577 | -.082 | .066 | .040 | .000 |
| Empathy 3 | -.191 | .593 | -.028 | -.065 | .122 | .133 | .621 | -.140 | .048 | .056 | .073 |
| Empathy 4 | .022 | .419 | .046 | -.029 | -.024 | .025 | .643 | .110 | .010 | .194 | .188 |
| Empathy 5 | -.105 | .616 | .150 | .026 | .114 | .029 | .622 | .045 | -.039 | .051 | .077 |
| Empathy 6 | -.133 | .680 | .060 | -.098 | .196 | .078 | .577 | -.142 | .005 | .080 | .062 |
| Empathy 7 | -.242 | .709 | .164 | -.227 | .069 | -.123 | .695 | -.047 | -.182 | .040 | -.056 |
| Empathy 8 | -.046 | .283 | .637 | .144 | .347 | .249 | .674 | -.052 | -.080 | .001 | .109 |
| Flexibility 1 | .711 | -.304 | .004 | .043 | -.146 | -.076 | -.013 | .655 | -.059 | .029 | -.110 |
| Flexibility 2 | .785 | -.307 | .095 | -.077 | .049 | -.005 | -.049 | .665 | -.038 | -.002 | -.171 |
| Flexibility 3 | .689 | -.323 | -.028 | .057 | -.010 | .003 | -.008 | .668 | .013 | .007 | -.127 |
| Flexibility 4 | .646 | -.209 | .030 | .063 | -.108 | -.245 | -.034 | .646 | .208 | -.037 | .119 |
| Flexibility 5 | .779 | .085 | .136 | .287 | .046 | .138 | .007 | .696 | .168 | -.018 | .259 |
| Flexibility 6 | .743 | .021 | .150 | .202 | .123 | .072 | .053 | .581 | .200 | .011 | .060 |
| Flexibility 7 | .475 | -.039 | .059 | -.208 | .184 | .110 | -.096 | .432 | .220 | .049 | .283 |
| Flexibility 8 | .718 | -.090 | .065 | .096 | .025 | -.085 | -.159 | .571 | .147 | .017 | .167 |
| Social In. 1 | -.041 | .012 | .202 | .058 | .071 | .721 | -.141 | -.320 | .135 | .337 | .322 |
| Social In. 2 | .131 | -.239 | .565 | -.048 | .142 | -.163 | .109 | .147 | .126 | .630 | -.028 |
| Social In. 3 | .009 | -.060 | .659 | .313 | .185 | -.047 | .192 | .091 | .217 | .765 | -.012 |
| Social In. 4 | .053 | .236 | .671 | .198 | .268 | .307 | .170 | -.144 | .030 | .584 | .366 |
| Social In. 5 | .132 | .176 | .567 | .118 | .000 | .209 | .039 | -.090 | .043 | .457 | .203 |
| Social In. 6 | .185 | .275 | .569 | .085 | .037 | .427 | .018 | -.220 | .001 | .475 | .356 |
| Social In. 7 | -.038 | .205 | .779 | .102 | .118 | .091 | .218 | -.040 | .140 | .781 | .087 |
| Social In. 8 | .200 | .108 | .598 | .252 | -.168 | -.156 | -.018 | .081 | .204 | .633 | -.043 |
| Stability 1 | .265 | -.024 | .006 | .773 | .013 | .121 | -.172 | .201 | .650 | .072 | .082 |
| Stability 2 | .142 | -.143 | .124 | .748 | .153 | .036 | -.066 | .071 | .734 | .014 | .099 |
| Stability 3 | -.005 | -.051 | .159 | .801 | .153 | -.192 | -.017 | .060 | .768 | .129 | .110 |
| Stability 4 | .000 | -.125 | .145 | .616 | -.010 | .133 | .081 | .010 | .525 | .161 | .051 |
| Stability 5 | .052 | .212 | -.073 | .291 | .590 | .046 | .085 | .041 | .399 | .045 | .160 |
| Stability 6 | .024 | -.028 | .266 | .493 | .255 | .171 | -.011 | .019 | .740 | .388 | -.019 |
| Stability 7 | .046 | -.068 | .246 | .705 | .132 | -.156 | -.025 | .123 | .612 | .147 | -.080 |
| Stability 8 | .021 | -.087 | -.001 | .272 | .537 | .197 | -.121 | .027 | .269 | -.036 | .168 |
| Openness 1 | .045 | .263 | .275 | -.017 | .779 | -.062 | .278 | .263 | .001 | .134 | .423 |
| Openness 2 | .014 | .284 | .297 | .046 | .699 | -.003 | .252 | -.066 | .127 | .039 | .571 |
| Openness 3 | .025 | .239 | .127 | .222 | .489 | -.030 | -.068 | .097 | .132 | .092 | .359 |
| Openness 4 | -.056 | .527 | .148 | -.027 | .505 | .153 | .116 | -.173 | .271 | .024 | .487 |
| Openness 5 | -.435 | -.047 | -.022 | -.018 | .398 | .384 | .058 | -.243 | .073 | .094 | .169 |
| Openness 6 | -.291 | .331 | .038 | -.138 | .205 | .320 | .417 | .018 | .051 | .078 | .300 |
| Openness 7 | .124 | .312 | .280 | -.324 | .263 | .115 | .393 | .119 | -.077 | .163 | .456 |
| Openness 8 | .007 | .454 | .197 | -.255 | .409 | .217 | .389 | .063 | .026 | .105 | .406 |

**Table A3d** Factor scores of each sample for the acculturation scale

|  | Hungary | | | Serbia | | | | Czech Republic | | | Germany | | |
| --- | --- | --- | --- | --- | --- | --- | --- | --- | --- | --- | --- | --- | --- |
|  | F1 | F2 | F3 | F1 | F2 | F3 | F4 | F1 | F2 | F3 | F1 | F2 | F3 |
| Int 1 | -.218 | .035 | .526 | .512 | -.010 | .325 | .600 | -.009 | -.113 | .285 | -.161 | .023 | .820 |
| Ass 1 | -.195 | .776 | .133 | -.142 | .124 | -.220 | .857 | -.007 | .776 | -.028 | .047 | .759 | -.017 |
| Sep 1 | .456 | .433 | -.215 | -.863 | -.136 | -.049 | .108 | .222 | .530 | -.007 | .785 | .180 | -.085 |
| Int 2 | .063 | .061 | .717 | .273 | .768 | -.038 | .020 | .371 | -.002 | .607 | -.073 | -.203 | .802 |
| Sep 2 | .642 | .101 | .023 | -.772 | -.064 | -.109 | -.016 | .639 | .119 | .179 | .906 | -.068 | .021 |
| Int 3 | -.142 | -.186 | .475 | -.275 | .778 | -.025 | .196 | .076 | -.066 | .786 | -.555 | -.198 | .374 |
| Ass 2 | .697 | .225 | -.118 | .434 | .526 | .276 | .112 | .528 | .463 | -.135 | .786 | .314 | -.282 |
| Ass 3 | .761 | -.038 | -.161 | .535 | .753 | .179 | -.072 | .727 | .187 | .109 | .277 | .715 | -.057 |
| Sep 3 | .824 | -.089 | -.137 | .670 | .435 | .223 | .263 | .868 | .146 | .081 | .925 | -.050 | .005 |
| Int 4 | .531 | .456 | -.192 | .586 | .604 | .191 | .079 | .414 | .518 | -.102 | .453 | .510 | -.176 |
| Ass 4 | .815 | .178 | -.267 | -.646 | -.615 | -.075 | .182 | .454 | .533 | -.138 | .768 | .372 | -.253 |
| Int 5 | -.565 | .375 | .463 | .208 | .180 | .831 | -.055 | -.022 | .098 | .447 | -.831 | .124 | .141 |
| Ass 5 | .361 | .548 | -.076 | .038 | -.030 | .852 | -.043 | .104 | .563 | .034 | -.234 | .764 | -.133 |

**Table A3e** Factor scores of each sample for the Multiethnic Identity Measure

|  | Hungary | | | | Serbia | | | Germany | | | |
| --- | --- | --- | --- | --- | --- | --- | --- | --- | --- | --- | --- |
|  | F1 | F2 | F3 | F4 | F1 | F2 | F3 | F1 | F2 | F3 | F4 |
| MeimEi 1 | .433 | -.034 | .214 | .405 | .394 | .743 | -.044 | .221 | .637 | .042 | .174 |
| MeimEi 2 | .413 | -.102 | .295 | .180 | .097 | .539 | -.374 | .302 | .164 | -.277 | .146 |
| MeimEi 3 | .474 | .219 | .223 | .346 | .320 | .624 | -.028 | .378 | .623 | .018 | .127 |
| MeimOu 1 | .187 | .703 | .282 | .105 | .038 | -.256 | .628 | -.017 | .108 | .695 | -.352 |
| MeimEi 4 | .063 | .021 | .345 | .094 | .018 | .666 | .018 | .069 | .633 | .178 | .007 |
| MeimEi 5 | .655 | .023 | .064 | .274 | .826 | .120 | -.139 | .785 | .127 | -.121 | .001 |
| MeimOu 2 | .057 | -.402 | .151 | .142 | .197 | .329 | -.706 | .161 | .137 | -.235 | .676 |
| MeimEi 6 | -.144 | -.010 | -.047 | -.474 | -.414 | -.404 | .107 | -.083 | -.473 | .259 | .465 |
| MeimOu 3 | -.047 | .490 | .499 | -.118 | .082 | .365 | .604 | -.063 | .043 | .828 | -.055 |
| MeimEi 7 | -.123 | .000 | .013 | -.724 | -.267 | -.619 | -.062 | -.019 | -.676 | .056 | .122 |
| MeimEi 8 | .818 | -.081 | .008 | .114 | .757 | .421 | -.067 | .811 | .254 | -.133 | .051 |
| MeimEi 9 | .566 | .140 | .297 | .198 | .467 | .558 | .125 | .399 | .542 | -.028 | -.035 |
| MeimEi 10 | .315 | .006 | .626 | .020 | .473 | .521 | -.036 | .235 | .679 | .190 | .266 |
| MeimEi 11 | .829 | .101 | .125 | .096 | .731 | .294 | -.131 | .797 | .181 | -.068 | .146 |
| MeimOu 4 | -.003 | -.564 | .104 | -.074 | .116 | .128 | -.693 | .152 | .072 | -.238 | .584 |
| MeimEi 12 | .633 | .036 | .164 | -.035 | .696 | .083 | .067 | .448 | .246 | .086 | .205 |
| MeimOu 5 | .061 | .606 | .569 | -.081 | .001 | .309 | .670 | -.027 | .072 | .817 | .101 |
| MeimEi 13 | .830 | -.047 | .025 | .137 | .785 | .383 | -.107 | .816 | .180 | -.113 | .179 |
| MeimOu 6 | .126 | .704 | .231 | .098 | -.339 | .101 | .409 | -.031 | .070 | .787 | -.206 |
| MeimEi 14 | .771 | .215 | .040 | .111 | .812 | .193 | -.105 | .786 | .033 | .117 | -.034 |

**Table A4** Item difficulties and sensitivities over all samples

|  | Hungary | | Czech Republic | | Serbia | | Germany | |
| --- | --- | --- | --- | --- | --- | --- | --- | --- |
|  | D | S | D | S | D | S | D | S |
| Multi ethnic identity measure |  |  |  |  |  |  |  |  |
| Ethnic Identity |  |  |  |  |  |  |  |  |
| MeimEi 1 | 63,41 | 0,76 | 55,41 | 0,68 | 71,1 | 0,52 | 68,06 | 0,7 |
| MeimEi 2 | 84,09 | 0,3 | 74,83 | 0,38 | 90,14 | 0,62 | 91,11 | 0,39 |
| MeimEi 3 | 44,86 | 0,62 | 41,72 | 0,6 | 44,4 | 0,53 | 57,17 | 0,72 |
| MeimEi 4 | 90,29 | 0,48 | 83,65 | 0,43 | 94,88 | 0,62 | 84,96 | 0,36 |
| MeimEi 5 | 50,34 | 0,76 | 47,33 | 0,7 | 46,89 | 0,61 | 55,12 | 0,66 |
| MeimEi 6 | 55,43 | 0,73 | 45,52 | 0,62 | 38,95 | 0,34 | 61,81 | 0,75 |
| MeimEi 7 | 63,1 | 0,59 | 52,1 | 0,58 | 46,32 | 0,77 | 59,58 | 0,56 |
| MeimEi 8 | 45,9 | 0,52 | 46,01 | 0,41 | 43,06 | 0,43 | 48,26 | 0,32 |
| MeimEi 9 | 67,82 | 0,62 | 59,57 | 0,56 | 54,83 | 0,63 | 54,45 | 0,66 |
| MeimEi 10 | 43,2 | 0,19 | 40,61 | 0,39 | 38,37 | 0,42 | 51,02 | 0,41 |
| MeimEi 11 | 62,5 | 0,69 | 67,79 | 0,46 | 56,08 | 0,7 | 62,46 | 0,65 |
| MeimEi 12 | 85,14 | 0,34 | 81,9 | 0,12 | 70,43 | 0,54 | 72,74 | 0,27 |
| MeimEi 13 | 86,9 | 0,36 | 65,56 | -0,92 | 67,66 | 0,57 | 75,01 | 0,4 |
| MeimEi 14 | 56,32 | 0,76 | 54,4 | 0,52 | 44,15 | 0,84 | 52,42 | 0,77 |
| MeimOu |  |  |  |  |  |  |  |  |
| MeimOu 1 | 59,88 | 0,67 | 53,1 | 0,74 | 50,05 | 0,67 | 56,96 | 0,6 |
| MeimOu 2 | 43,58 | 0,46 | 36,81 | 0,42 | 41,05 | 0,68 | 45,39 | 0,6 |
| MeimOu 3 | 56,98 | 0,8 | 51,98 | 0,68 | 51,29 | 0,74 | 51,16 | 0,71 |
| MeimOu 4 | 56,21 | 0,59 | 57,67 | 0,53 | 44,02 | 0,55 | 55,98 | 0,48 |
| MeimOu 5 | 54,94 | 0,77 | 52,03 | 0,78 | 47,73 | 0,84 | 49,02 | 0,75 |
| MeimOu 6 | 62,76 | 0,74 | 65,43 | 0,43 | 53,97 | 0,73 | 64,1 | 0,57 |
| Cultural Intelligence Scale |  |  |  |  |  |  |  |  |
| Metacognitive 1 | 82,9 | 0,88 | 80,22 | 0,75 | 80,22 | 0,76 | 79,58 | 0,71 |
| Metacognitive 2 | 87,37 | 0,86 | 86,12 | 0,69 | 86,12 | 0,82 | 79,59 | 0,72 |
| Metacognitive 3 | 84,47 | 0,91 | 84,69 | 0,82 | 84,69 | 0,85 | 77,69 | 0,8 |
| Metacognitive 4 | 83,16 | 0,8 | 85,9 | 0,66 | 85,9 | 0,78 | 76,43 | 0,71 |
| Metacognitive 5 | 55,76 | 0,66 | 57,65 | 0,52 | 57,65 | 0,59 | 61,5 | 0,67 |
| Metacognitive 6 | 63,42 | 0,75 | 77,59 | 0,22 | 77,59 | 0,6 | 73,58 | 0,57 |
| Cognitive 1 | 66,75 | 0,91 | 85,42 | 0,78 | 85,42 | 0,83 | 76,94 | 0,77 |
| Cognitive 2 | 60,82 | 0,84 | 74,12 | 0,81 | 74,12 | 0,77 | 63,6 | 0,75 |
| Cognitive 3 | 65,71 | 0,77 | 78,07 | 0,77 | 78,07 | 0,76 | 48,69 | 0,59 |
| Cognitive 4 | 58,64 | 0,83 | 66,35 | 0,68 | 66,35 | 0,82 | 59,93 | 0,7 |
| Motivational 1 | 87,72 | 0,71 | 74,18 | 0,73 | 98,24 | 0,77 | 87,6 | 0,79 |
| Motivational 2 | 75,65 | 0,88 | 73,93 | 0,71 | 96,63 | 0,77 | 86,77 | 0,77 |
| Motivational 3 | 75,83 | 0,87 | 71,67 | 0,72 | 84,86 | 0,72 | 86,05 | 0,81 |
| Motivational 4 | 61,84 | 0,83 | 57,45 | 0,74 | 86,86 | 0,83 | 73,71 | 0,79 |
| Motivational 5 | 74,43 | 0,73 | 72,41 | 0,74 | 88,92 | 0,69 | 85,78 | 0,71 |
| Behavioral 1 | 68,59 | 0,8 | 65,6 | 0,61 | 68,03 | 0,77 | 81,34 | 0,67 |
| Behavioral 2 | 64,9 | 0,82 | 56,81 | 0,75 | 65,06 | 0,85 | 68,09 | 0,68 |
| Behavioral 3 | 70,26 | 0,88 | 63,33 | 0,8 | 67,71 | 0,91 | 90,29 | 0,69 |
| Behavioral 4 | 65,97 | 0,89 | 64,82 | 0,79 | 68,59 | 0,79 | 83,33 | 0,82 |
| Behavioral 5 | 62,22 | 0,88 | 62,2 | 0,74 | 61,08 | 0,83 | 76,18 | 0,78 |
| Multicultural Personality Questionnaire | | | |  |  |  |  |  |
| Empathy 1 | 74,43 | 0,82 | 66,31 | 0,6 | 74,38 | 0,81 | 72,29 | 0,75 |
| Empathy 2 | 75,04 | 0,81 | 71,24 | 0,72 | 75,7 | 0,7 | 70,37 | 0,58 |
| Empathy 3 | 75,04 | 0,53 | 70,03 | 0,58 | 75,87 | 0,66 | 68,65 | 0,64 |
| Empathy 4 | 65,17 | 0,73 | 58,66 | 0,61 | 65,35 | 0,4 | 68,52 | 0,65 |
| Empathy 5 | 72,07 | 0,79 | 68,24 | 0,56 | 67,82 | 0,63 | 71,45 | 0,62 |
| Empathy 6 | 70,28 | 0,71 | 63,64 | 0,63 | 72,22 | 0,75 | 66,62 | 0,6 |
| Empathy 7 | 72,34 | 0,83 | 63,06 | 0,59 | 73,15 | 0,75 | 71,16 | 0,69 |
| Empathy 8 | 69,84 | 0,73 | 61,69 | 0,51 | 63,96 | 0,4 | 68,89 | 0,69 |
| Flexibility 1 | 59,88 | 0,72 | 50 | 0,51 | 62,81 | 0,77 | 53,69 | 0,6 |
| Flexibility 2 | 64,62 | 0,72 | 57,44 | 0,66 | 61,49 | 0,84 | 60,4 | 0,64 |
| Flexibility 3 | 57,09 | 0,74 | 48,64 | 0,72 | 63,79 | 0,76 | 49,15 | 0,65 |
| Flexibility 4 | 67,11 | 0,79 | 53,15 | 0,64 | 60,86 | 0,66 | 58,5 | 0,7 |
| Flexibility 5 | 57,14 | 0,7 | 43,85 | 0,46 | 48,69 | 0,75 | 56,76 | 0,76 |
| Flexibility 6 | 63,84 | 0,65 | 52,93 | 0,45 | 49,92 | 0,75 | 58,86 | 0,59 |
| Flexibility 7 | 65,6 | 0,59 | 64,66 | 0,34 | 51,4 | 0,44 | 64,91 | 0,51 |
| Flexibility 8 | 61,46 | 0,68 | 58,76 | 0,5 | 62,15 | 0,73 | 60,27 | 0,6 |
| Social Initiative 1 | 58,82 | 0,58 | 51,6 | 0,45 | 45,57 | 0,63 | 52,14 | 0,56 |
| Social Initiative 2 | 50,98 | 0,11 | 48,13 | -0,05 | 51,16 | 0,00 | 50,57 | 0,09 |
| Social Initiative 3 | 40,21 | 0,09 | 43,25 | -0,11 | 38,18 | -0,16 | 40,21 | -0,1 |
| Social Initiative 4 | 60,11 | 0,59 | 49,43 | 0,52 | 58,87 | 0,6 | 61,7 | 0,45 |
| Social Initiative 5 | 57,41 | 0,7 | 51,53 | 0,28 | 63,18 | 0,53 | 63,3 | 0,48 |
| Social Initiative 6 | 56,61 | 0,7 | 50,72 | 0,58 | 53,69 | 0,76 | 57,36 | 0,66 |
| Social Initiative 7 | 60,41 | 0,48 | 53,75 | 0,15 | 59,7 | 0,44 | 60,45 | 0,19 |
| Social Initiative 8 | 45,12 | 0,14 | 44,42 | 0,11 | 43,84 | -0,12 | 49,72 | -0,07 |
| Emotional Stability 1 | 56,26 | 0,71 | 43,99 | 0,64 | 60,67 | 0,72 | 60,15 | 0,65 |
| Emotional Stability 2 | 46,37 | 0,67 | 46,81 | 0,38 | 52,71 | 0,74 | 47,24 | 0,7 |
| Emotional Stability 3 | 58,33 | 0,7 | 47,6 | 0,61 | 48,93 | 0,75 | 49,81 | 0,77 |
| Emotional Stability 4 | 46,9 | 0,62 | 44,88 | 0,54 | 52,22 | 0,66 | 44,75 | 0,55 |
| Emotional Stability 5 | 47,5 | -0,05 | 49,05 | -0,38 | 56,4 | -0,15 | 53,8 | -0,38 |
| Emotional Stability 6 | 45,5 | 0,73 | 49,35 | 0,44 | 47,29 | 0,52 | 46,38 | 0,79 |
| Emotional Stability 7 | 47,16 | 0,63 | 52,9 | 0,35 | 53,28 | 0,72 | 51,52 | 0,62 |
| Emotional Stability 8 | 46,03 | -0,07 | 48,18 | 0,00 | 46,35 | -0,19 | 47,1 | -0,23 |
| Openness 1 | 59,7 | 0,69 | 54,71 | 0,62 | 64,12 | 0,79 | 67,62 | 0,55 |
| Openness 2 | 64,11 | 0,75 | 58,81 | 0,65 | 66,09 | 0,76 | 63,05 | 0,63 |
| Openness 3 | 52,38 | 0,69 | 44,54 | 0,63 | 54,68 | 0,51 | 48,19 | 0,33 |
| Openness 4 | 67,91 | 0,75 | 55,6 | 0,51 | 68,8 | 0,76 | 63,49 | 0,41 |
| Openness 5 | 56,15 | 0,74 | 46,01 | 0,57 | 50,33 | 0,38 | 39,05 | 0,22 |
| Openness 6 | 61,44 | 0,74 | 55,07 | 0,53 | 66,25 | 0,44 | 58,74 | 0,54 |
| Openness 7 | 65,6 | 0,6 | 49,71 | 0,55 | 59,36 | 0,48 | 57,1 | 0,59 |
| Openness 8 | 70,02 | 0,65 | 58,44 | 0,53 | 74,54 | 0,66 | 66,48 | 0,59 |
| Acculturation scale |  |  |  |  |  |  |  |  |
| Integration 1 | 52,19 | 0,54 | 59,93 | 0,28 | 55,07 | 0,45 | 58,87 | 0,61 |
| Assimilation 1 | 56,4 | 0,67 | 60,85 | 0,67 | 56,12 | 0,64 | 58,45 | 0,52 |
| Separation 1 | 54,14 | 0,42 | 54,42 | 0,66 | 56,6 | 0,37 | 46,73 | 0,59 |
| Integration 2 | 59,44 | -0,33 | 48,15 | -0,01 | 57,97 | 0,79 | 65,41 | -0,6 |
| Separation 2 | 45,5 | 0,52 | 44,75 | 0,4 | 50,64 | 0,42 | 34,03 | 0,48 |
| Integration 3 | 49,91 | 0,11 | 33,33 | 0,49 | 50,32 | -0,05 | 55,34 | 0,51 |
| Assimilation 2 | 63,39 | 0,73 | 54,96 | 0,71 | 55,66 | 0,84 | 73,1 | 0,73 |
| Assimilation 3 | 68,52 | 0,63 | 64,96 | 0,53 | 61,27 | 0,73 | 60,09 | 0,66 |
| Separation 3 | 65,86 | 0,8 | 51,42 | 0,72 | 53,46 | -0,79 | 73,4 | 0,75 |
| Integration 4 | 57,45 | 0,62 | 38,01 | 0,55 | 51,13 | 0,14 | 46,76 | 0,42 |
| Assimilation4 | 61,46 | 0,53 | 45,32 | 0,36 | 53,7 | 0,86 | 75,65 | 0,73 |
| Integration 5 | 63,86 | 0,7 | 62,91 | 0,68 | 57 | 0,9 | 83,57 | 0,9 |
| Assimilation5 | 70,99 | 0,66 | 67,02 | 0,7 | 57,8 | -1,18 | 84,4 | 0,9 |
| Blatant and subtle Prejudice |  |  |  |  |  |  |  |  |
| Threat | 70,11 | 0,61 | 61,04 | 0,65 | 67,32 | 0,87 | 86,47 | 0,79 |
| Threat | 59,65 | 0,66 | 40,75 | 0,61 | 59,2 | 0,7 | 70,4 | 0,7 |
| Threat | 70,77 | 0,66 | 60,61 | 0,68 | 69,21 | 0,8 | 85,4 | 0,76 |
| Threat | 47,43 | 0,13 | 46,74 | 0,66 | 54,35 | 0,18 | 73,69 | 0,8 |
| Threat | 72,96 | 0,46 | 52,35 | 0,54 | 70,36 | 0,22 | 85,35 | 0,86 |
| Threat | 39,87 | 0,32 | 36,71 | 0,2 |  | 0,87 | 48,68 | 0,51 |
| Differences | 61,94 | 0,61 | 48,64 | 0,57 |  |  | 73,91 | 0,57 |
| Differences | 53,67 | 0,66 | 37,77 | 0,58 |  |  | 58,48 | 0,71 |
| Differences | 57,62 | 0,76 | 45,99 | 0,72 |  |  | 76,06 | 0,8 |
| Differences | 57,62 | 0,61 | 50,94 | 0,59 |  |  | 63,79 | 0,7 |
| Intimacy | 36,36 | 0,7 | 50,29 | 0,67 |  |  | 35,74 | 0,71 |
| Intimacy | 48,12 | 0,7 | 51,58 | 0,67 |  |  | 41,05 | 0,71 |
| Intimacy | 33,77 | 0,68 | 30,65 | 0,63 |  |  | 38,43 | 0,63 |
| Intimacy | 24,87 | 0,81 | 24,57 | 0,64 |  |  | 29,43 | 0,73 |
| Positive Emotions | 30,72 | 0,76 | 32,61 | 0,6 |  |  | 32,31 | 0,66 |
| Positive Emotions | 22,82 | 0,6 | 24,09 | 0,57 |  |  | 28,54 | 0,5 |

**Notes.** D = Difficulty, S = selectivity.
